# Nucleophilic C4-selective (hetero) arylation of pyridines for facile synthesis of heterobiaryls

**DOI:** 10.3389/fchem.2023.1254632

**Published:** 2023-09-01

**Authors:** Kewon Kim, Euna You, Sungwoo Hong

**Affiliations:** ^1^ Department of Chemistry, Korea Advanced Institute of Science and Technology (KAIST), Daejeon, Republic of Korea; ^2^ Center for Catalytic Hydrocarbon Functionalizations, Institute for Basic Science (IBS), Daejeon, Republic of Korea

**Keywords:** C-H (hetero) arylation, pyridinium salt, C4-selectivity, heterobiaryl, metal-free

## Abstract

The synthesis of heterobiaryl compounds holds significant value in organic chemistry due to their extensive range of applications. Herein, we report a highly efficient strategy for conducting C4-selective (hetero) arylation of pyridines using *N*-aminopyridinium salts. The reaction proceeds readily at room temperature in the presence of a base, thus eliminating the requirement for catalysts or oxidants. This method allows for the installation of various electron-rich (hetero) aryl groups on pyridines, resulting in the streamlined synthesis of highly valuable C4-(hetero) aryl pyridine derivatives, which are otherwise challenging to acquire via conventional methods. This simple and straightforward method will facilitate access to a range of heterobiaryl compounds thereby promoting their application in various scientific disciplines.

## Introduction

**SCHEME 1:**
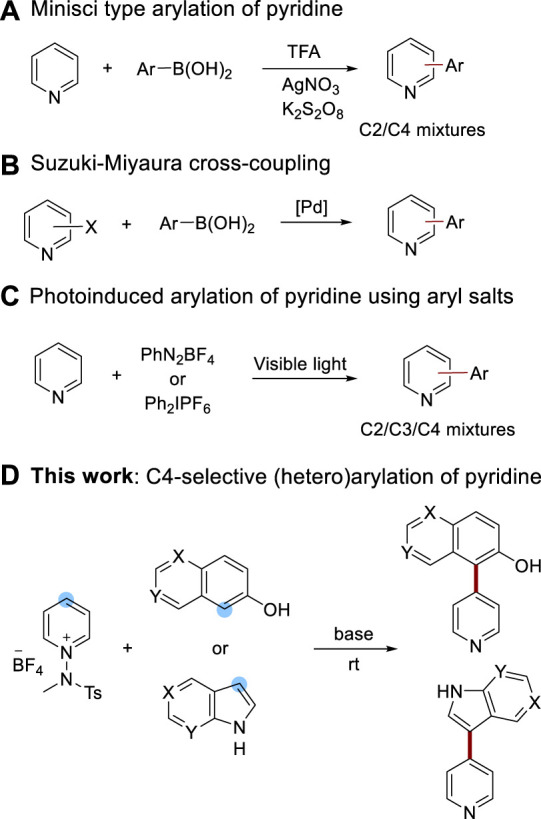
Synthetic approaches to (hetero) arylation of pyridine cores.

Pyridine, a prominent component in the realms of agrochemicals, pharmaceuticals, and functional materials (Vitaku et al., 2014; Zhong, et al., 2014), is often a key constituent of *N*-heterobiaryl scaffolds due to their rigid and adaptable three-dimensional structures (Wu and Chan, 2006; Ghoteimi et al., 2023; Yang et al., 2016; Lee, H. et al., 2019; Jo, W. et al., 2020; Li, H. et al., 2021; Shao et al., 2021; Nguyen, N. H. et al., 2022; Takahashi, F., and Yorimitsu, H., 2023). These structures are frequently used in the fabrication of therapeutic agents or as ligands for metal catalyst complexes. For instance, LJH685, a selective RSK inhibitor, has been designed to treat triple-negative breast cancer (TNBC) (Cui et al., 2022), and Etoricoxib (Chauret et al., 2001), a selective cyclooxygenase-2 inhibitor, has been developed for its anti-inflammatory effects. As a result, the synthesis of arylated pyridine scaffolds has become a hot topic, with a majority of strategies employing transition metal catalysts (Li et al., 2014; Lee, D. et al., 2015; Zuo et al., 2015; Jiao et al., 2016; Lutz et al., 2016; Jia et al., 2018; Gu, X. et al., 2022). The Baran group, for example, introduced a Minisci-type Ag-catalyzed arylation of pyridine and other heteroarenes (Seiph et al., 2010) (Scheme 1A). In addition, modified versions of the Suzuki-Miyaura reaction using heteroaryl halides and Pd catalysts have been reported (Ichikawa et al., 2017) (Scheme 1B). However, despite their synthetic versatility, these methods demand high temperatures and exhibit limited functional group tolerance. The advent of photocatalyzed reactions utilizing diazonium salts (Bartolomeu et al., 2019) and diaryliodonium salts (Tobisu et al., 2013) as aryl radical precursors marked a significant advance (Scheme 1C). Although these reactions are achievable at room temperature, the poor site selectivity of pyridine continues to pose a challenge.

Recently, pyridinium salts have emerged as versatile synthetic tools for pyridine functionalization, delivering mild conditions and superior site selectivity (Kim et al., 2021; Shin et al., 2021; Choi et al., 2022; Kim et al., 2022a; Kim et al., 2022b; Kim et al., 2022c). As pyridine surrogates, *N*-substituted pyridinium salts have bolstered the development of numerous visible-light induced reactions where radicals are produced via photocatalysts or electron donor-acceptor (EDA) complex formations. These radicals initiate a hydrogen atom transfer (HAT) or react with unsaturated carbon species, giving rise to complex radical species that can be sequestered in the pyridinium salts (Kim et al., 2019; Lee et al., 2020; Mathi et al., 2020). Of particular interest is the site-selective functionalization controlled by the *N*-substituents attached to the pyridinium salts (Jung et al., 2019). Beyond radical pathways, two-electron pathways have also been investigated, with pyridine or other heteroarene salts acting as electrophiles, demonstrating impressive reactivity and site selectivity (Okamoto et al., 1963; Okamoto et al., 1966; Lemire et al., 2004; Donohoe et al., 2009; Chupakhin et al., 2017). While one reference exists on pyridine C4 selective arylation using Grignard reagents, there are inherent compatibility challenges with various functional groups during the arylation process. As a result, the substrate scope is constrained, with only a single example reported (Katritzky and Beltrami, 1979). Despite these advances, the direct cross-coupling of pyridinium salts with other (hetero) arenes under mild conditions is still an underexplored field. Motivated by the versatile reactivity and excellent selectivity of *N*-substituted pyridinium salts, we aimed to design a method for C4-selective (hetero) arylation of pyridine under mild reaction conditions. We hypothesized that electron-rich (hetero) arenes, such as indoles and naphthol, would undergo nucleophilic addition to electrophilic pyridinium salts, leading to the formation of *N*-(hetero) biaryl compounds incorporating pyridine. This new approach enables the synthesis of a variety of *N*-(hetero) biaryl building blocks, known to be invaluable scaffolds in the sphere of medicinal chemistry (Marchais-Oberwinkler et al., 2008; Guo et al., 2011; Tsuji, T. et al., 2020; Yuan et al., 2020; Chaudhary, 2021).

## Result and discussion

First, we conducted a comprehensive screening of reaction conditions to facilitate the incorporation of an indole moiety onto pyridine, employing pyridinium salt **1a** and 5-methoxyindole **2a**. We assessed the efficiency of various organic and inorganic bases (Table 1, entries 1–5). Cs_2_CO_3_ failed to show any significant conversion, while DBU and NaO*t*Bu generated moderate yields. Notably, alongside the desired product **3b**, we also detected minor side product **N1**, which exhibited a bond formation between the nitrogen of the indole and the C4 position of the pyridine. Specifically, side product **3b′** resulted from an additional nucleophilic addition of the free N–H from the desired product, **3b**, to another pyridinium salt. When a stronger base, NaH, was used, the yield of the desired product **3b** was slightly improved. Prior research has suggested that the choice of base cation can affect the reactivity and selectivity (Kobayashi et al., 2015; Chen and Wu, 2017). To investigate the potential influence of base cations on our reaction, we further scrutinized various *tert*-butoxide species with differing cations, due to the similar conversion but milder basicity of NaO*t*Bu in comparison with NaH (entries 5 and 6). Remarkably, LiO*t*Bu yielded a result similar to NaO*t*Bu but demonstrated significantly superior selectivity for the indole C3 position. Subsequently, we explored the effects of ratio of **2a** and LiO*t*Bu (entries 7–13). A lower base quantity resulted in a decreased yield, and it was concluded that an optimal condition involved the use of 2.0 equiv of base (entry 10). When 2.0 equiv of **2a** was used, the yield significantly improved (entry 13). This enhancement can be attributed to the unfavorable formation of **3b′** in the presence of excess **2a**. Additionally, we evaluated other solvents with varying polarities, but DMSO proved to be superior (entries 14 and 15). Without the base, the reaction did not proceed (entry 16). Considering these optimization results, we selected entry 13 as the optimal condition for the introduction of the indole group at the pyridine C4 position.

**TABLE 1:** Optimization of reaction conditions.^a^

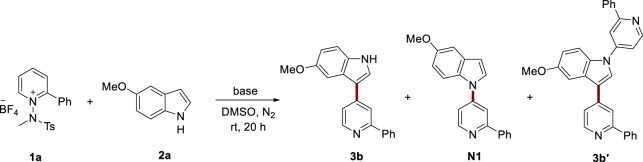				

Entry	**1a** (equiv)	**2a** (equiv)	Base (equiv)	Yield^b^ (**3b/N1/3b′**) (%)
1	1.5	1.0	Cs_2_CO_3_ (2.0)	trace/trace/trace
2	1.5	1.0	NaO*t*Bu (2.0)	48/17/9
3	1.5	1.0	DBU (2.0)	38/trace/trace
4	1.5	1.0	NaH (2.0)	51/13/12
5	1.5	1.0	KO*t*Bu (2.0)	26/9/5
6	1.5	1.0	LiO*t*Bu (2.0)	48/3/7
7	1.0	1.0	LiO*t*Bu (1.2)	46/6/4
8	1.0	1.0	LiO*t*Bu (1.5)	49/5/4
9	1.0	1.0	LiO*t*Bu (1.8)	55/5/5
10	1.0	1.0	LiO*t*Bu (2.0)	57/5/4
11	1.0	1.0	LiO*t*Bu (2.5)	55/4/4
12	1.0	1.5	LiO*t*Bu (2.0)	73/8/trace
**13**	**1.0**	**2.0**	**LiO*t*Bu (2.0)**	**82/8/trace**
14^c^	1.0	2.0	LiO*t*Bu (2.0)	18/2/trace
15^d^	1.0	2.0	LiO*t*Bu (2.0)	trace/trace/trace
16	1.0	2.0	no base	trace/trace/trace

Entry 13 was selected as an optimal condition.

^a^ Reaction conditions: **1a** or **2a** was used as a limiting reagent (0.1 mmol for 1.0 equiv) in DMSO (1.0 mL) under N_2_ atmosphere at rt for 20 h.

^b^ NMR, yields were measured with the caffeine as an internal standard.

^c^ DMF, was used instead of DMSO.

^d^ THF, was used instead of DMSO.

Utilizing the optimized conditions for heteroarylations, we explored the scope of the reaction by including various functional groups on indoles (Table 2). The reactions showed consistent performance across a variety of indoles, from simple indoles to those substituted with electron-donating groups, such as methoxy, and methyl groups (**3a**–**3e**). Various halogen groups, spanning from iodo to fluoro, resulted in satisfactory conversions in the reaction (**3f**–**3i**). Electron-withdrawing groups, including nitro (**3j**), cyano (**3k**), amide (**3l**), and aldehyde (**3m**) groups, were successfully accommodated under the reaction conditions, leading to good conversions. Remarkably, even in the presence of a strong base, the hydroxyl group (**3n**) yielded a significant output. We also tested azaindole (**3o**) and pyrrolopyrimidine (**3p**), both of which are common derivatives of indoles found in medicinal chemistry. Pleasingly, the reactions proceeded well, highlighting the applicability of this method for the synthesis of these vital indole derivatives.

**TABLE 2:** Nucleophilic heteroarylation of pyridine with indoles.^a^

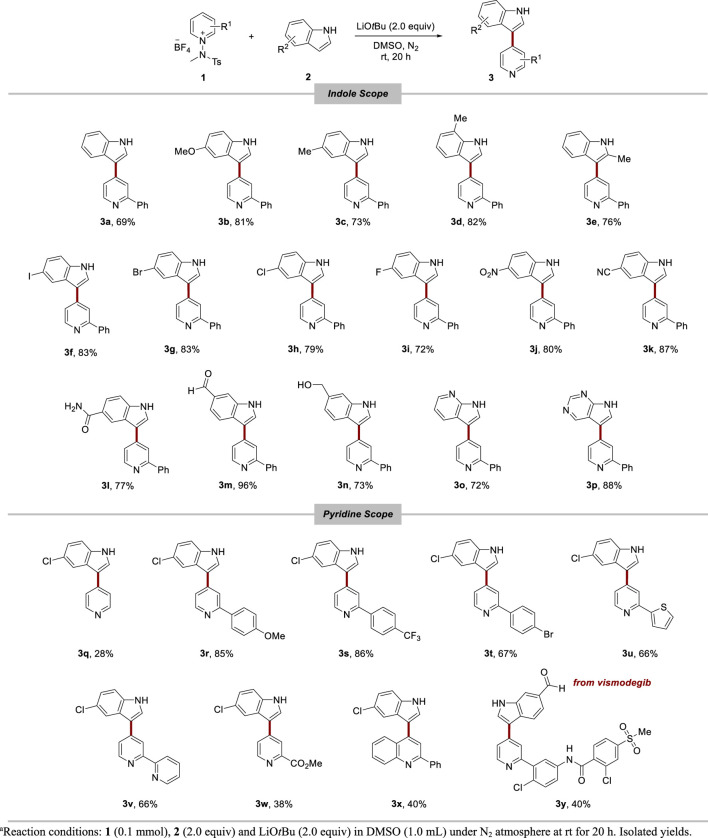

Subsequently, we systematically evaluated a series of pyridinium salts with diverse substituents to examine their reactivity and suitability with the established reaction conditions. The utilization of unsubstituted pyridinium salts resulted in comparatively lower yields (**3q**) than when 2-substituted pyridinium salts were used. When pyridinium salts carried an aryl substituent, the reaction progressed efficiently, suggesting that the aryl group present on the pyridinium salts enhances the reaction (**3r**–**3t**). The incorporation of other aryl groups, such as thiophene (**3u**) and pyridine (**3v**), into the pyridinium salt successfully yielded the corresponding products. We found that the reaction proceeded successfully when using C2-ester pyridinium salt **3w**. Additionally, we expanded the applicability of our method by facilitating reactions with quinolinium salt (**3x**). When we used a pyridinium salt derived from a pharmaceutical compound, vismodegib, the reaction produced the desired product **3y** in 40% yield.

We discovered that 2-naphthol could be effectively installed at the C4 position of pyridine in the presence of a base. Following the screening of reaction conditions using pyridinium salt **1a** and 2-naphthol **4a** as model substrates, we identified an optimized reaction condition that led to high conversion yields towards compound **5a** (see Supplementary Table S1 for details). In the naphthol scope (Table 3), we noted a promising tolerance towards a variety of functional groups. The reaction progressed smoothly for substrates containing electron-donating groups such as methyl and methoxy groups (**5a**–**5e**). Halogen groups, including bromo (**5f**), chloro (**5g**), and fluoro (**5h**), were also compatible with the reaction conditions. Furthermore, electron-withdrawing groups, like cyano, amide, ester, ketone, and aldehyde groups, resulted in excellent conversion (**5i**–**5m**). The nucleophilic alcohol group also showed favorable conversion (**5n**). Motivated by this broad functional group tolerance, we extended our investigation to other heteroarenes as naphthol derivatives (**5o**–**5r**). Pleasingly, the reactions involving quinoline (**5o**), isoquinoline (**5p**), and benzothiophene (**5r**) proceeded well. However, benzofuran (**5q**) exhibited lower compatibility with the reaction conditions. Within the pyridine scope, improved conversion was observed with pyridine salts bearing a phenyl group compared to simple pyridine salts (**5s**–**5v**). Pyridinium salts substituted with thiophene (**5w**) and pyridine (**5x**) resulted in high conversions. Pleasingly, excellent yields were observed with the pyridinium salt carrying an ester group (**5y**) and the quinolinium salt (**5z**). Furthermore, a promising conversion was demonstrated when using the pyridinium salt derived from vismodegib (**5aa**), indicating the efficiency of this method in introducing a naphthol moiety even in the late stage of complex, pyridine-containing molecules.

**TABLE 3:** Nucleophilic arylation of pyridine with 2-naphthols.^a^

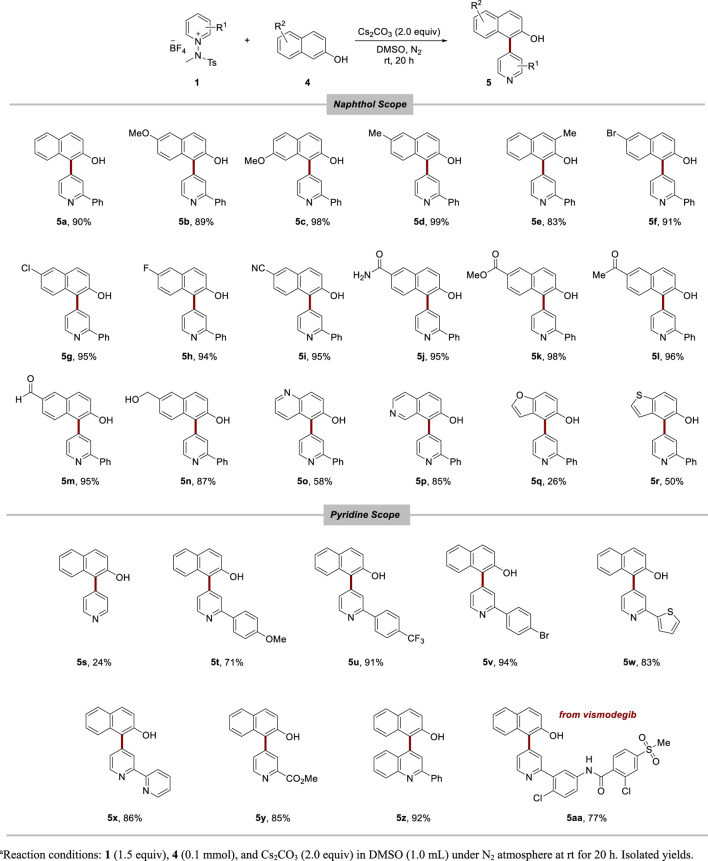

**SCHEME 2:**
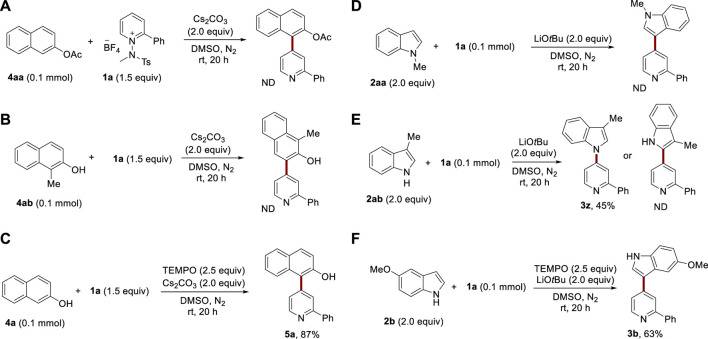
Mechanistic studies.

To further elucidate the reaction mechanism, we conducted several control experiments. When the hydroxyl group of 2-naphthol was protected with an acyl group, the desired product was not observed, implying that the deprotonation of the hydroxyl group is a critical step (Scheme 2A). Blocking the C1 position of 2-naphthol with a methyl group halted the reaction. This evidence, together with the synthesis of **5e**, distinctly indicates that under the optimal reaction conditions, the most reactive site of 2-naphthol towards the pyridinium salt is the C1 position (Scheme 2B). In the context of indoles, when *N*-methylated indole was used as a substrate, no reaction was observed, highlighting that a free N–H is vital for the reaction to proceed (Scheme 2D). When the C3 position of indole was blocked, the only significant product observed was **3z**, which resulted from the nucleophilic attack of nitrogen from **2ab** on the pyridinium salt (Scheme 2E). The addition of a radical scavenger, (2,2,6,6-tetramethylpiperidine-1-oxyl) (TEMPO) did not inhibit reactivity, suggesting that the reaction proceeded via an ionic pathway (Schemes 2C, F).

**SCHEME 3:**
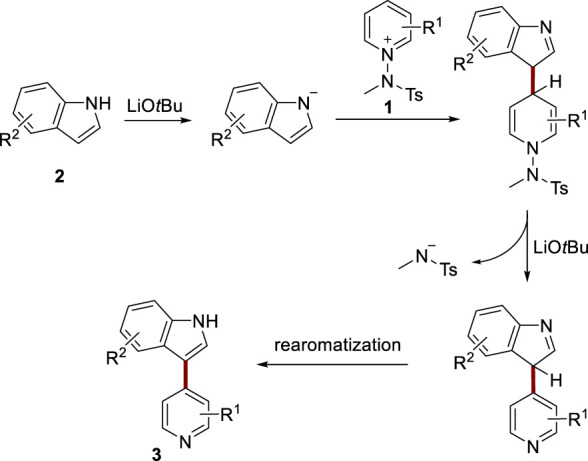
Proposed reaction mechanisms.

Given the experimental evidence, a plausible reaction mechanism for the arylation of pyridine with indole is depicted in Scheme 3. The reaction commences with the base-induced deprotonation of indole, followed by nucleophilic addition to the pyridinium salt **1**. This addition is succeeded by the base-assisted aromatization of the pyridine moiety, leading to the release of a tosyl amine anion. Intriguingly, this anionic tosyl amine could potentially function as a base for the rearomatization of indole. Upon completion of the reaction, the reaction mixture is subjected to mild acidic workup, leading to the formation of the desired product **3**. A similar pathway is expected for naphthol and is outlined in Supplementary Scheme S1.

## Conclusion

In summary, we have developed a mild and selective nucleophilic (hetero) arylation of pyridine with exclusive C4 selectivity at the pyridine ring. This method employs *N*-aminopyridinium salts as electrophiles, enabling the facile and efficient synthesis of (hetero) arylated pyridine derivatives incorporating a wide range of aromatic functionalities. With the assistance of a base, indoles, naphthols, and their respective heteroarene derivatives can undergo nucleophilic addition onto the pyridine, eliminating the need for additional protection or deprotection steps. Interestingly, these nucleophilic (hetero) arenes exhibit unique and selective reactivity when engaging with the pyridinium salt. This selective behavior allows for meticulous control over the regiochemistry of the arylation reaction, facilitating the introduction of (hetero) aryl groups at the C4 position of pyridine. Given the observed extensive functional group tolerance, the developed protocols have the potential to yield a vast array of heterobiaryl scaffolds, which are indispensable building blocks in medicinal chemistry. These insights will contribute to the ongoing evolution of synthetic methods, simultaneously unlocking new possibilities for the discovery and development of innovative pharmaceutical compounds.

## Data Availability

The original contributions presented in the study are included in the article/Supplementary Material, further inquiries can be directed to the corresponding author.
