# Supplementary material for: Nucleophilic C4-selective (hetero) arylation of pyridines for facile synthesis of heterobiaryls
Source: Front Chem. 2023 Sep 1;11:1254632. doi: 10.3389/fchem.2023.1254632 (PMC10502421; doi:10.3389/fchem.2023.1254632)
Supplement: Supplementary file 1 [file DataSheet1.docx]

Supplementary Material

Nucleophilic C4-Selective Arylation of Pyridines for Facile Synthesis of Heterobiarenes.

Kewon Kim, Euna You, Sungwoo Hong*

Department of Chemistry, Korea Advanced Institute of Science and Technology (KAIST) & Center for Catalytic Hydrocarbon Functionalizations, Daejeon, Korea

*** Correspondence:** Sungwoo Hong: hongorg@kaist.ac.kr

**Table of Contents**

1. **General Information**
2. **Study for Naphthol Introduction on Pyridine**
3. **Experimental Procedure**
4. **Characterization of Compounds**

***Appendix I***

**Spectral Copies of 1H-, 13C-Data**

**1. General Information**

Unless stated otherwise, reactions were performed in flame-dried glassware. Analytical thin layer chromatography (TLC) was performed on precoated silica gel 60 F^254^ plates, and visualization of TLC was achieved by light (254 and 365 nm). Flash column chromatography was performed on silica gel (400-630 mesh) or a Combi*Flash*^®^ *R_f_^+^* system with Redi*Sep*^®^ *R_f_* silica columns (230-400 mesh) using a proper eluent. ^1^H NMR was recored on Brucker Advance 400MHz, 500MHz or Agilent Technologies DD2 600MHz. Chemical shifts were quoted in parts per million (ppm) referenced to the appropriate solvent peak or 0.0 ppm for tetramethylsilane.a The following abbreviations were used to describe peak splitting patterns when appropriate: br = broad, s = singlet, d = doublet, t = triplet, q = quartet, p = pentet, m = multiplet, dd = doublet, dt = doublet of triplet, dq = doublet of quartet, td = triplet of doublet, tt = triplet of triplet, ddd = doublet of doublet of doublet, dtd = doublet of triplet of doublet. Coupling constants, *J*, were reported in hertz unit (Hz). ^13^C NMR was recorded on Bruker Avance 100 MHz, and was fully decoupled by broad band proton decoupling. Chemical shifts were reported in ppm referenced to the centerline of a quintet at 53.8 ppm of Methylene Chloride-*d*_2_, septet at 49.0 ppm of MeOD-*d*_4_ or a septet at 39.5 ppm of DMSO-*d*_6_. High resolution mass spectrometry was conducted on an ESI from KBSI (Ochang). Commercial grade reagents and solvents were used without further purification except as indicated below.

**2. Study for Naphthol Introduction on Pyridine**

| **Table S1** \| Optimization of reaction condition – introduction of naphthol on pyridine.^a^ | | | | |
| --- | --- | --- | --- | --- |
|  | | | | |
| **Entry** | **Base** | **Solvent** | **Temp (°C)** | **Yield (%)^b^** |
| **1** | **Cs_2_CO_3_** | **DMSO** | **rt** | **95** |
| 2 | K_2_CO_3_ | DMSO | rt | 83 |
| 3 | NaO*t*Bu | DMSO | rt | 73 |
| 4 | NaOH | DMSO | rt | 10 |
| 5 | K_3_PO_4_ | DMSO | rt | 15 |
| 6 | DBU | DMSO | rt | 79 |
| 7 | TEA | DMSO | rt | Trace |
| 8 | DABCO | DMSO | rt | Trace |
| 9 | None | DMSO | rt | Trace |
| 10 | Cs_2_CO_3_ | DCE | rt | 17 |
| 11 | Cs_2_CO_3_ | Dioxane | rt | Trace |
| 12 | Cs_2_CO_3_ | DCM | rt | 15 |
| 13 | Cs_2_CO_3_ | Acetonitrile | rt | 45 |
| 14 | Cs_2_CO_3_ | DMF | rt | 79 |
| 15 | Cs_2_CO_3_ | Acetone | rt | 11 |
| 16 | Cs_2_CO_3_ | EtOH | rt | Trace |
| 17 | Cs_2_CO_3_ | MeOH | rt | Trace |
| 18 | Cs_2_CO_3_ | DMSO | 40 | 80 |
| 19 | Cs_2_CO_3_ | DMSO | 60 | 70 |
| 20 | Cs_2_CO_3_ | DMSO | 80 | 38 |
| 21^c^ | Cs_2_CO_3_ | DMSO | rt | 59 |
| 22^d^ | Cs_2_CO_3_ | DMSO | rt | 78 |
| 23^e^ | Cs_2_CO_3_ | DMSO | rt | 88 |
| ^a^Reaction conditions: **4a** (0.1 mmol), **1a** (1.5 equiv), and base (2.0 equiv) in DMSO (1.0 mL) under N_2_ atmosphere at rt for 20 h.  ^b^NMR yield  ^c^**1a**(0.1 mmol), **4a** (1.0 equiv), Cs_2_CO_3_ (2.0 equiv) were used  ^d^**1a** (0.1 mmol), **4a** (1.5 equiv), Cs_2_CO_3_ (2.0 equiv) were used  ^e^**1a** (0.1 mmol), **4a** (2.0 equiv), Cs_2_CO_3_ (2.0 equiv) were used | | | | |

|  |
| --- |
| **Scheme S1**. Proposed reaction mechanism. |

**3. Experiment Procedure**

**3.1 General experimental procedure for introduction of indoles on pyridine**

In a 15 mL test tube with a stirring bar, **1** (0.1 mmol), **2** (2.0 equiv), and LiO*t*Bu (2.0 equiv) were added. The test tube was then evacuated and backfilled with N_2_, followed by adding DMSO (1.0 mL). The mixture was stirred for 20 h at room temperature. After the reaction was completed, it was quenched with saturated NH_4_Cl solution (15 mL), and then the diethyl ether (15 mL X 3) was added for extraction. The organic layer was dried over with Na_2_SO_4_, filtered, and concentrated under reduced pressure. The crude mixture was purified by flash column chromatography on silica gel to afford the desired product **3**.

**3.2 General experimental procedure for introduction of naphthol on pyridine**

In a 15 mL test tube with a stirring bar, **4** (0.1 mmol), **1** (1.5 equiv), and Cs_2_CO_3_ (2.0 equiv) were added. The test tube was then evacuated and backfilled with N_2_, followed by adding DMSO (1.0 ml). The mixture was stirred for 20 h at room temperature. After the reaction was completed, it was quenched with saturated NH_4_Cl solution (15 mL), and then the diethyl ether (15 mL X 3) was added for extraction. The organic layer was dried over with Na_2_SO_4_, filtered, and concentrated under reduced pressure. The crude mixture was purified by flash column chromatography on silica gel to afford the desired product **5**.

**4. Characterization of Compounds**

For rotameric mixtures, pairs of resonances are represented to two decimal places, and minor rotamer are enclosed in parenthesis.

**3-(2-phenylpyridin-4-yl)-1H-indole (3a)**. (18.6 mg, 69%). ^1^H NMR (400 MHz, DMSO-*d*_6_) δ 11.74 (s, 1H), 8.64 (d, *J* = 5.2 Hz, 1H), 8.22 – 8.19 (m, 2H), 8.19 – 8.15 (m, 2H), 8.07 – 8.02 (m, 1H), 7.72 (dd, *J* = 5.2, 1.7 Hz, 1H), 7.57 – 7.49 (m, 3H), 7.49 – 7.38 (m, 1H), 7.26 – 7.15 (m, 2H). ^13^C NMR (100 MHz, DMSO-*d*_6_) δ 156.4, 149.8, 144.5, 139.1, (137.19), 137.02, 128.9, 128.7, 126.7, (126.42), 126.25, (124.67), 124.64, 121.9, 120.5, 119.3, 116.8, (112.93), 112.90, (112.33), 112.27. MS-ESI (m/z) calcd. for [C_19_H_15_N_2_]^+^ : 271.1235, found : 271.1235.

**5-methoxy-3-(2-phenylpyridin-4-yl)-1H-indole (3b)**. (24.2 mg, 81%). ^1^H NMR (400 MHz, Methylene Chloride-*d*_2_) δ 8.68 (dd, *J* = 5.1, 0.8 Hz, 1H), 8.61 (br, 1H), 8.12 – 8.07 (m, 2H), 8.04 (dd, *J* = 1.8, 0.8 Hz, 1H), 7.63 (d, *J* = 2.8 Hz, 1H), 7.55 (dd, *J* = 5.2, 1.7 Hz, 1H), 7.53 – 7.47 (m, 2H), 7.47 – 7.42 (m, 2H), 7.40 (d, *J* = 8.8 Hz, 1H), 6.95 (dd, *J* = 8.9, 2.4 Hz, 1H), 3.88 (s, 3H). ^13^C NMR (100 MHz, Methylene Chloride-*d*_2_) δ 158.0, 155.7, 150.4, 144.7, 140.2, 132.4, 129.2, 129.1, 127.3, 126.0, 124.8, 120.4, 118.5, 115.6, 113.2, 112.9, 101.9, 56.2. MS-ESI (m/z) calcd. for [C_20_H_17_N_2_O]^+^ : 301.1341, found : 301.1342.

**5-methyl-3-(2-phenylpyridin-4-yl)-1H-indole (3c)**. (21.0 mg, 73%). ^1^H NMR (400 MHz, DMSO-*d*_6_) δ 11.62 (s, 1H), 8.63 (d, *J* = 5.2 Hz, 1H), 8.20 – 8.16 (m, 3H), 8.14 (d, *J* = 2.8 Hz, 1H), 7.82 (s, 1H), 7.72 (dd, *J* = 5.2, 1.7 Hz, 1H), 7.55 – 7.49 (m, 2H), 7.48 – 7.42 (m, 1H), 7.39 (d, *J* = 8.2 Hz, 1H), 7.04 (dd, *J* = 8.4, 1.6 Hz, 1H), 2.46 (s, 3H). ^13^C NMR (100 MHz, DMSO-*d*_6_) δ 156.2, 149.8, 144.7, 139.1, 135.5, 129.1, 128.9, 128.7, 126.7, 126.5, 124.9, 123.5, 119.2, 118.9, 116.8, 112.5, 112.0, 21.4. MS-ESI (m/z) calcd. for [C_20_H_17_N_2_]^+^ : 285.1392, found : 285.1392.

**7-methyl-3-(2-phenylpyridin-4-yl)-1H-indole (3d)**. (23.1 mg, 82%). ^1^H NMR (400 MHz, DMSO-*d*_6_) δ 11.70 (s, 1H), 8.63 (d, *J* = 5.2 Hz, 1H), 8.23 – 8.15 (m, 4H), 7.87 (d, *J* = 8.0 Hz, 1H), 7.73 (dd, *J* = 5.2, 1.7 Hz, 1H), 7.53 (dd, *J* = 8.2, 6.5 Hz, 2H), 7.49 – 7.43 (m, 1H), 7.10 (t, *J* = 7.6 Hz, 1H), 7.02 (d, *J* = 7.1 Hz, 1H), 2.52 (s, 3H). ^13^C NMR (100 MHz, DMSO-*d*_6_) δ 156.3, 149.7, 144.7, 139.0, 136.68, (136.52), 128.9, 128.7, 126.7, 126.26, (126.09), 124.42, (124.39), 122.5, 121.55, (121.50), 120.7, 119.3, 116.9, 113.32, (113.28), 16.8. MS-ESI (m/z) calcd. for [C_20_H_17_N_2_]^+^ : 285.1392, found : 285.1391.

**2-methyl-3-(2-phenylpyridin-4-yl)-1H-indole (3e)**. (22.0 mg, 76%). ^1^H NMR (400 MHz, DMSO-*d*_6_) δ 11.54 (s, 1H), 8.69 (d, *J* = 5.2 Hz, 1H), 8.14 – 8.09 (m, 2H), 8.00 (d, *J* = 1.6 Hz, 1H), 7.72 (dd, *J* = 7.4, 1.5 Hz, 1H), 7.57 – 7.51 (m, 3H), 7.50 – 7.45 (m, 1H), 7.42 – 7.38 (m, 1H), 7.14 (td, *J* = 7.9, 7.5, 1.4 Hz, 1H), 7.09 (td, *J* = 7.4, 1.4 Hz, 1H), 2.61 (s, 3H). ^13^C NMR (100 MHz, DMSO-*d*_6_) δ 156.3, 149.6, 144.6, 139.0, 135.42, (135.26), 134.93, (134.78), 129.0, 128.8, 126.7, 126.39, (126.35), 121.9, 121.2, 120.0, 119.4, 117.7, 111.14, (111.09), 109.98, (109.95), 12.77. MS-ESI (m/z) calcd. for [C_20_H_17_N_2_]^+^ : 285.1392, found : 285.1392.

**5-iodo-3-(2-phenylpyridin-4-yl)-1H-indole (3f)**. (31.0 mg, 83%). ^1^H NMR (400 MHz, DMSO-*d*_6_) δ 11.92 (s, 1H), 8.66 (d, *J* = 5.2 Hz, 1H), 8.31 (s, 1H), 8.22 – 8.15 (m, 4H), 7.69 (dd, *J* = 5.2, 1.7 Hz, 1H), 7.57 – 7.42 (m, 4H), 7.37 (d, *J* = 8.5 Hz, 1H). ^13^C NMR (100 MHz, DMSO-*d*_6_) δ 156.4, 150.0, 143.7, 139.0, 136.2, 130.0, 129.0, 128.7, 127.4, 127.4, 127.3, 126.7, 119.5, 117.1, 114.8, 112.5, 84.6. MS-ESI (m/z) calcd. for [C_19_H_14_IN_2_]^+^ : 397.0202, found : 397.0202.

**5-bromo-3-(2-phenylpyridin-4-yl)-1H-indole (3g)**. (29.4 mg, 83%). ^1^H NMR (400 MHz, DMSO-*d*_6_) δ 11.95 (s, 1H), 8.65 (d, *J* = 5.2 Hz, 1H), 8.26 (d, *J* = 2.8 Hz, 1H), 8.19 – 8.16 (m, 3H), 8.14 (d, *J* = 1.9 Hz, 1H), 7.70 (dd, *J* = 5.2, 1.7 Hz, 1H), 7.56 – 7.42 (m, 4H), 7.34 (dd, *J* = 8.6, 1.9 Hz, 1H). ^13^C NMR (100 MHz, DMSO-*d*_6_) δ 156.4, 150.0, 143.7, 139.0, 135.9, 129.0, 129.7, 127.9, 126.7, 126.4, 124.5, 121.4, 119.4, 117.0, 114.3, 113.1, 112.8. MS-ESI (m/z) calcd. for [C_19_H_14_BrN_2_]^+^ : 349.0340, found : 349.0340.

**5-chloro-3-(2-phenylpyridin-4-yl)-1H-indole (3h)**. (24.2 mg, 79%). ^1^H NMR (400 MHz, DMSO-*d*_6_) δ 11.94 (s, 1H), 8.65 (d, *J* = 5.2 Hz, 1H), 8.28 (d, *J* = 2.8 Hz, 1H), 8.21 – 8.15 (m, 3H), 8.01 (d, *J* = 2.0 Hz, 1H), 7.71 (dd, *J* = 5.2, 1.7 Hz, 1H), 7.55 – 7.49 (m, 3H), 7.48 – 7.43 (m, 1H), 7.23 (dd, *J* = 8.7, 2.0 Hz, 1H). ^13^C NMR (100 MHz, DMSO-*d*_6_) δ 156.4, 150.0, 143.7, 139.0, 135.7, 129.0, 128.7, 128.1, 126.8, 125.7, 125.1, 122.0, 119.4, 118.5, 117.0, 113.9, 112.9. MS-ESI (m/z) calcd. for [C_19_H_14_ClN_2_]^+^ : 305.0846, found : 305.0847.

**5-fluoro-3-(2-phenylpyridin-4-yl)-1H-indole (3i)**. (21.0 mg, 72%). ^1^H NMR (400 MHz, DMSO-*d*_6_) δ 11.86 (s, 1H), 8.64 (d, *J* = 5.2 Hz, 1H), 8.28 (d, *J* = 2.8 Hz, 1H), 8.23 – 8.12 (m, 3H), 7.77 (dd, *J* = 10.5, 2.5 Hz, 1H), 7.70 (dd, *J* = 5.2, 1.7 Hz, 1H), 7.56 – 7.48 (m, 3H), 7.48 – 7.42 (m, 1H), 7.07 (td, *J* = 9.1, 2.5 Hz, 1H). ^13^C NMR (100 MHz, DMSO-*d*_6_) δ 157.8 (d, *J* = 232.8 Hz), 156.4, 149.9, 144.0, 139.1, 133.8, 128.9, 128.7, 128.3, 126.8, 124.8 (d, *J* = 10.0 Hz), 119.2, 116.7, 113.4 (d, *J* = 9.9 Hz), 113.2 (d, *J* = 4.6 Hz), 110.1 (d, *J* = 26.0 Hz), 104.3 (d, *J* = 24.2 Hz). MS-ESI (m/z) calcd. for [C_19_H_14_FN_2_]^+^ : 289.1141, found : 289.1142.

**5-nitro-3-(2-phenylpyridin-4-yl)-1H-indole (3j)**. (25.1 mg, 80%). ^1^H NMR (400 MHz, DMSO-*d*_6_) δ 12.43 (s, 1H), 8.87 (d, *J* = 2.2 Hz, 1H), 8.73 (dd, *J* = 5.1, 0.7 Hz, 1H), 8.44 (d, *J* = 2.7 Hz, 1H), 8.25 (dd, *J* = 1.7, 0.8 Hz, 1H), 8.23 – 8.16 (m, 2H), 8.12 (dd, *J* = 9.0, 2.2 Hz, 1H), 7.76 (dd, *J* = 5.1, 1.7 Hz, 1H), 7.70 (d, *J* = 9.0 Hz, 1H), 7.58 – 7.50 (m, 2H), 7.52 – 7.43 (m, 1H). ^13^C NMR (100 MHz, DMSO-*d*_6_) δ 156.6, 150.2, 142.9, 141.7, 140.2, 138.8, 130.0, 129.1, 128.7, 126.7, 124.0, 119.8, 117.5, 117.3, 116.2, 115.6, 112.9. MS-ESI (m/z) calcd. for [C_19_H_14_N_3_O_2_]^+^ : 316.1086, found : 316.1087.

**3-(2-phenylpyridin-4-yl)-1H-indole-5-carbonitrile (3k)**. (26.0 mg, 87%). ^1^H NMR (400 MHz, DMSO-*d*_6_) δ 12.27 (s, 1H), 8.67 (dd, *J* = 5.2, 0.7 Hz, 1H), 8.56 (d, *J* = 1.4 Hz, 1H), 8.40 (d, *J* = 2.7 Hz, 1H), 8.26 – 8.17 (m, 3H), 7.79 (dd, *J* = 5.2, 1.7 Hz, 1H), 7.67 (d, *J* = 8.5 Hz, 1H), 7.57 (dd, *J* = 8.4, 1.5 Hz, 1H), 7.57 – 7.48 (m, 2H), 7.51 – 7.42 (m, 1H). ^13^C NMR (100 MHz, DMSO-*d*_6_) δ 156.5, 150.0, 143.1, 138.9, 138.9, 129.0, 128.9, 128.7, 126.8, 125.1, 124.8, 124.4, 120.6, 119.8, 117.3, 114.1, 113.6, 102.6. MS-ESI (m/z) calcd. for [C_20_H_14_N_3_]^+^ : 296.1188, found : 296.1189.

**3-(2-phenylpyridin-4-yl)-1H-indole-5-carboxamide (3l)**. (24.3 mg, 77%). ^1^H NMR (400 MHz, DMSO-*d*_6_) δ 11.94 (s, 1H), 8.69 (d, *J* = 5.2 Hz, 1H), 8.57 (d, *J* = 1.5 Hz, 1H), 8.27 (d, *J* = 2.5 Hz, 1H), 8.24 (d, *J* = 1.7 Hz, 1H), 8.23 – 8.16 (m, 2H), 8.07 (s, 1H), 7.82 (dd, *J* = 5.2, 1.7 Hz, 1H), 7.78 (dd, *J* = 8.5, 1.6 Hz, 1H), 7.57 – 7.49 (m, 3H), 7.50 – 7.42 (m, 1H), 7.21 (s, 1H). ^13^C NMR (100 MHz, DMSO-*d*_6_) δ 168.88, (168.83), 156.4, 149.9, 144.0, 139.04, 138.7, (138.6), 129.0, 128.7, 127.66, (127.48), 126.8, 124.08, (124.05), 121.9, 119.7, 119.3, 117.2, 114.07, (114.04), 111.76, (111.70). MS-ESI (m/z) calcd. for [C_20_H_16_N_3_O]^+^ : 314.1293, found : 314.1292.

**3-(2-phenylpyridin-4-yl)-1H-indole-6-carbaldehyde (3m)**. (29.1 mg, 96%). ^1^H NMR (400 MHz, DMSO-*d*_6_) δ 12.32 (s, 1H), 10.07 (s, 1H), 8.68 (d, *J* = 5.1 Hz, 1H), 8.50 (s, 1H), 8.24 (d, *J* = 1.6 Hz, 1H), 8.22 – 8.17 (m, 3H), 8.09 (d, *J* = 1.4 Hz, 1H), 7.76 (dd, *J* = 5.2, 1.7 Hz, 1H), 7.72 (dd, *J* = 8.4, 1.4 Hz, 1H), 7.53 (dd, *J* = 8.2, 6.5 Hz, 2H), 7.49 – 7.43 (m, 1H). ^13^C NMR (100 MHz, DMSO-*d*_6_) δ 192.7, 156.5, 149.9, 143.6, 138.9, 136.6, 131.0, 130.8, 129.1, 129.1, 128.7, 126.8, 120.3, 119.8, 119.6, 117.2, 116.1, 113.9. MS-ESI (m/z) calcd. for [C_20_H_15_N_2_O]^+^ : 299.1184, found : 299.1184.

**(3-(2-phenylpyridin-4-yl)-1H-indol-6-yl)methanol (3n)**. (23.2 mg, 73%). ^1^H NMR (400 MHz, DMSO-*d*_6_) δ 11.68 (s, 1H), 8.63 (d, *J* = 5.2 Hz, 1H), 8.23 – 8.14 (m, 4H), 7.98 (d, *J* = 8.3 Hz, 1H), 7.71 (dd, *J* = 5.2, 1.6 Hz, 1H), 7.57 – 7.48 (m, 2H), 7.50 – 7.41 (m, 2H), 7.14 (dd, *J* = 8.3, 1.5 Hz, 1H), 5.17 (t, *J* = 5.7 Hz, 1H), 4.62 (d, *J* = 4.7 Hz, 2H). ^13^C NMR (100 MHz, DMSO-*d*_6_) δ 156.3, 149.8, 144.6, 139.1, 137.35, (137.19), 136.6, 128.9, 128.7, 126.7, 126.26, (126.09), 123.56, (123.52), 119.8, 119.1, 118.9, 116.7, 112.81, (112.78), 110.09, (110.03), 63.3. MS-ESI (m/z) calcd. for [C_20_H_17_N_2_O]^+^ : 301.1341, found : 301.1342.

**3-(2-phenylpyridin-4-yl)-1H-pyrrolo[2,3-b]pyridine (3o)**. (26.6 mg, 72%). ^1^H NMR (400 MHz, DMSO-*d*_6_) δ 12.27 (s, 1H), 8.65 (d, *J* = 5.2 Hz, 1H), 8.49 (dd, *J* = 8.1, 1.5 Hz, 1H), 8.39 (d, *J* = 2.7 Hz, 1H), 8.33 (dd, *J* = 4.6, 1.5 Hz, 1H), 8.25 (d, *J* = 1.6 Hz, 1H), 8.22 – 8.16 (m, 2H), 7.75 (dd, *J* = 5.2, 1.7 Hz, 1H), 7.52 (t, *J* = 7.3 Hz, 2H), 7.48 – 7.42 (m, 1H), 7.24 (dd, *J* = 8.0, 4.6 Hz, 1H). ^13^C NMR (100 MHz, DMSO-*d*_6_) δ 156.5, 149.9, 149.3, 143.7, 143.4, 139.0, 129.0, 128.7, 128.0, 126.8, 126.8, 119.1, 117.1, 116.7, 116.6, 111.6. MS-ESI (m/z) calcd. for [C_18_H_14_N_3_]^+^ : 272.1188, found : 272.1189.

**5-(2-phenylpyridin-4-yl)-7H-pyrrolo[2,3-d]pyrimidine (3p)**. (18.0 mg, 88%). ^1^H NMR (400 MHz, DMSO-*d*_6_) δ 12.70 (s, 1H), 9.55 (s, 1H), 8.88 (s, 1H), 8.68 (d, *J* = 5.2 Hz, 1H), 8.50 (d, *J* = 2.7 Hz, 1H), 8.31 (d, *J* = 1.6 Hz, 1H), 8.27 – 8.20 (m, 2H), 7.82 (dd, *J* = 5.2, 1.7 Hz, 1H), 7.52 (dd, *J* = 8.2, 6.4 Hz, 2H), 7.49 – 7.43 (m, 1H). ^13^C NMR (100 MHz, DMSO-*d*_6_) δ 157.2, 152.7, 152.1, 150.6, 149.6, 142.8, 139.3, 129.5, 129.1, 128.0, 127.9, 127.3, 112.0, 117.3, 116.2, 112.4. MS-ESI (m/z) calcd. for [C_17_H_13_N_4_]^+^ : 273.1140, found : 273.1140.

**5-chloro-3-(pyridin-4-yl)-1H-indole (3q)**. (5.9 mg, 28%). ^1^H NMR (400 MHz, DMSO-*d*_6_) δ 11.92 (s, 1H), 8.55 (d, *J* = 5.3 Hz, 2H), 8.14 (d, *J* = 2.8 Hz, 1H), 8.00 (d, *J* = 2.0 Hz, 1H), 7.75 (d, *J* = 6.2 Hz, 2H), 7.52 (d, *J* = 8.6 Hz, 1H), 7.23 (dd, *J* = 8.6, 2.0 Hz, 1H). ^13^C NMR (100 MHz, DMSO-*d*_6_) δ 149.6, 143.0, 135.7, 127.9, 125.6, 125.2, 122.1, 120.6, 118.5, 113.9, 112.5. MS-ESI (m/z) calcd. for [C_13_H_10_ClN_2_]^+^ : 229.0533, found : 229.0532.

**5-chloro-3-(2-(4-methoxyphenyl)pyridin-4-yl)-1H-indole (3r)**. (28.0 mg, 85%). ^1^H NMR (400 MHz, DMSO-*d*_6_) δ 11.94 (s, 1H), 8.60 (d, *J* = 5.2 Hz, 1H), 8.27 (s, 1H), 8.16 – 8.12 (m, 2H), 8.12 – 8.11 (m, 1H), 8.00 (d, *J* = 2.0 Hz, 1H), 7.65 (dd, *J* = 5.3, 1.7 Hz, 1H), 7.53 (d, *J* = 8.6 Hz, 1H), 7.22 (dd, *J* = 8.6, 2.0 Hz, 1H), 7.11 – 7.02 (m, 2H), 3.84 (s, 3H). ^13^C NMR (100 MHz, DMSO-*d*_6_) δ 160.2, 156.0, 149.5, 143.8, 135.65, (135.48), 131.2, 128.1, 127.9, 125.73, (125.69), 125.1, 122.0, 118.7, 118.5, 116.2, 114.1, 113.86, (113.81), 112.95, (112.92), 55.2. MS-ESI (m/z) calcd. for [C_20_H_16_ClN_2_O]^+^ : 335.0951, found : 335.0951.

**5-chloro-3-(2-(4-(trifluoromethyl)phenyl)pyridin-4-yl)-1H-indole (3s)**. (32.0 mg, 86%). ^1^H NMR (400 MHz, DMSO-*d*_6_) δ 11.97 (s, 1H), 8.70 (d, *J* = 5.2 Hz, 1H), 8.41 (d, *J* = 8.1 Hz, 2H), 8.31 (s, 1H), 8.30 (d, *J* = 1.5 Hz, 1H), 8.04 (d, *J* = 2.0 Hz, 1H), 7.88 (d, *J* = 8.2 Hz, 2H), 7.79 (dd, *J* = 5.2, 1.7 Hz, 1H), 7.53 (d, *J* = 8.7 Hz, 1H), 7.23 (dd, *J* = 8.7, 2.0 Hz, 1H). ^13^C NMR (100 MHz, DMSO-*d*_6_) δ 154.8, 150.2, 144.0, 142.9, 135.7, 135.5, 129.08 (q, *J* = 31.5 Hz), (128.35), 128.17, 127.5, (125.65 (q, *J* = 3.7 Hz)), 125.55 (q, *J* = 4.1 Hz), 125.2, 124.4 (q, *J* = 271.7 Hz), 122.1, 120.2, 118.5, 117.7, (113.89), 113.84, (112.68), 112.65. MS-ESI (m/z) calcd. for [C_20_H_13_ClF_3_N_2_]^+^ : 373.0719, found : 373.0719.

**3-(2-(4-bromophenyl)pyridin-4-yl)-5-chloro-1H-indole (3t).** (26.3 mg, 67%). ^1^H NMR (400 MHz, DMSO-*d*_6_) δ 11.97 (s, 1H), 8.65 (d, *J* = 5.2 Hz, 1H), 8.29 (s, 1H), 8.21 (d, *J* = 1.6 Hz, 1H), 8.15 (d, *J* = 8.6 Hz, 2H), 8.02 (d, *J* = 2.0 Hz, 1H), 7.76 – 7.73 (m, 1H), 7.72 (d, *J* = 8.6 Hz, 2H), 7.53 (d, *J* = 8.6 Hz, 1H), 7.23 (dd, *J* = 8.6, 2.0 Hz, 1H). ^13^C NMR (100 MHz, DMSO-*d*_6_) δ 155.1, 149.9, 144.0, 138.1, (135.66), 135.49, 131.6, 128.8, (128.31), 128.13, (125.68), 125.64, 125.2, 122.6, 122.0, 119.7, 118.5, 117.0, (113.87), 113.82, (112.75), 112.72. MS-ESI (m/z) calcd. for [C_19_H_13_BrClN_2_]^+^ : 382.9951, found : 382.9951.

**5-chloro-3-(2-(thiophen-2-yl)pyridin-4-yl)-1H-indole (3u)**. (20.8 mg, 66%). ^1^H NMR (400 MHz, DMSO-*d*_6_) δ 11.96 (s, 1H), 8.50 (d, *J* = 5.2 Hz, 1H), 8.25 (s, 1H), 8.16 (d, *J* = 1.6 Hz, 1H), 8.01 (d, *J* = 2.0 Hz, 1H), 7.95 (dd, *J* = 3.7, 1.2 Hz, 1H), 7.64 (td, *J* = 5.2, 4.8, 1.4 Hz, 2H), 7.53 (d, *J* = 8.6 Hz, 1H), 7.28 – 7.17 (m, 2H). ^13^C NMR (100 MHz, DMSO-*d*_6_) δ 152.0, 149.7, 144.7, 143.8, 135.65, (135.48), 128.3, 128.23, (128.19), 128.0, 125.66, (125.63), 125.4, 125.2, 122.0, 119.2, 118.5, 115.1, 113.87, (113.82), 112.59, (112.56). MS-ESI (m/z) calcd. for [C_17_H_12_ClN_2_S]^+^ : 311.0410, found : 311.0410.

**3-([2,2'-bipyridin]-4-yl)-5-chloro-1H-indole (3v)**. (20.1 mg, 66%). ^1^H NMR (400 MHz, Methylene Chloride-*d*_2_) δ 8.77 (d, *J* = 5.4 Hz, 1H), 8.69 (d, *J* = 4.6 Hz, 1H), 8.68 – 8.63 (m, 1H), 8.51 (d, *J* = 8.0 Hz, 1H), 7.88 (td, *J* = 7.8, 1.8 Hz, 1H), 7.74 (d, *J* = 8.8 Hz, 1H), 7.67 (d, *J* = 2.1 Hz, 1H), 7.59 (d, *J* = 3.4 Hz, 1H), 7.49 (dd, *J* = 5.4, 2.2 Hz, 1H), 7.38 (dd, *J* = 7.8, 4.6 Hz, 1H), 7.26 (dd, *J* = 8.8, 2.1 Hz, 1H), 6.74 (dd, *J* = 3.4, 0.8 Hz, 1H). ^13^C NMR (100 MHz, Methylene Chloride-*d*_2_) δ 158.6, 158.6, 155.6, 151.2, 149.6, 147.7, 137.4, 134.0, 131.8, 128.8, 127.1, 124.7, 123.6, 121.5, 117.5, 114.7, 112.5, 105.5. MS-ESI (m/z) calcd. for [C_18_H_13_ClN_3_]^+^ : 306.0798, found : 306.0797.

**methyl 4-(5-chloro-1H-indol-3-yl)picolinate (3w)**. White solid (11.2 mg, 38%). ^1^H NMR (400 MHz, DMSO-*d*_6_) δ 12.03 (s, 1H), 8.66 (d, *J* = 5.2 Hz, 1H), 8.32 – 8.24 (m, 2H), 8.01 – 7.94 (m, 2H), 7.53 (d, *J* = 8.7 Hz, 1H), 7.24 (dd, *J* = 8.7, 2.0 Hz, 1H), 3.92 (s, 3H). ^13^C NMR (100 MHz, DMSO-*d*_6_) δ 165.5, 150.1, 147.9, 144.0, 135.75, (135.58), 128.61, (128.43), 125.49, (125.46), 125.4, 123.4, 122.2, 121.2, 118.2, 114.05, (114.00), 111.82, (111.79), 52.5. MS-ESI (m/z) calcd. for [C_15_H_12_ClN_2_O_2_]^+^ : 287.0582, found : 287.0587.

**4-(5-chloro-1H-indol-3-yl)-2-phenylquinoline (3x)**. Pale yellow solid (13.7 mg, 40%). ^1^H NMR (400 MHz, DMSO-*d*_6_) δ 12.01 (s, 1H), 8.34 – 8.28 (m, 2H), 8.18 (d, *J* = 8.4 Hz, 1H), 8.13 (s, 1H), 8.09 (d, *J* = 8.4 Hz, 1H), 8.00 (s, 1H), 7.84 (ddd, *J* = 8.3, 6.7, 1.4 Hz, 1H), 7.64 – 7.50 (m, 5H), 7.47 (d, *J* = 2.0 Hz, 1H), 7.24 (dd, *J* = 8.7, 2.1 Hz, 1H). ^13^C NMR (100 MHz, DMSO-*d*_6_) δ 168.3, 162.6, 155.7, 135.2, 134.9, 130.1, 129.7, 128.9, 127.5, 127.4, 126.5, 125.9, 125.6, 124.7, 122.0, 118.9, 118.2, 113.9, 111.7. MS-ESI (m/z) calcd. for [C_23_H_16_ClN_2_]^+^ : 355.0997, found : 355.1002.

**2-chloro-N-(4-chloro-3-(4-(6-formyl-1H-indol-3-yl)pyridin-2-yl)phenyl)-4-(methylsulfonyl)benzamide (3y)**. (23.4 mg, 40%). ^1^H NMR (400 MHz, DMSO-*d*_6_) δ 12.33 (d, *J* = 2.9 Hz, 1H), 10.95 (s, 1H), 10.06 (s, 1H), 8.71 (d, *J* = 5.2 Hz, 1H), 8.42 (s, 1H), 8.17 (d, *J* = 8.4 Hz, 1H), 8.14 (d, *J* = 1.7 Hz, 1H), 8.09 (d, *J* = 2.3 Hz, 2H), 8.04 – 7.99 (m, 2H), 7.92 (d, *J* = 8.0 Hz, 1H), 7.83 (dd, *J* = 5.3, 1.7 Hz, 1H), 7.79 (dd, *J* = 8.7, 2.7 Hz, 1H), 7.71 (dd, *J* = 8.4, 1.4 Hz, 1H), 7.63 (d, *J* = 8.7 Hz, 1H), 3.35 (s, 3H). ^13^C NMR (100 MHz, DMSO-*d*_6_) δ 192.7, 163.8, 156.2, 149.8, 143.2, 142.8, 140.8, 139.4, 137.5, 136.7, 136.5, 131.0, 130.8, 130.8, 130.4, 130.0, (129.05), 129.01, 128.1, 126.0, 122.4, 121.3, 120.9, 120.4, 120.0, 119.5, (116.13), 116.07, (113.48), 113.45, 43.1. MS-ESI (m/z) calcd. for [C_28_H_20_C_l2_N_3_O_4_S]^+^ : 564.0552, found : 564.0553.

**3-methyl-1-(2-phenylpyridin-4-yl)-1H-indole (3z)**. (13.0 mg, 45%). ^1^H NMR (400 MHz, Methanol-*d*_4_) δ 8.65 (d, *J* = 5.5 Hz, 1H), 8.02 – 7.96 (m, 2H), 7.94 (d, *J* = 2.1 Hz, 1H), 7.79 (dd, *J* = 8.3, 0.9 Hz, 1H), 7.63 – 7.56 (m, 2H), 7.55 – 7.43 (m, 4H), 7.29 (ddd, *J* = 8.4, 7.1, 1.3 Hz, 1H), 7.21 (ddd, *J* = 8.0, 7.1, 0.9 Hz, 1H), 2.37 (d, *J* = 1.2 Hz, 3H). ^13^C NMR (100 MHz, Methanol-*d*_4_) δ 160.8, 151.7, 149.6, 140.1, 132.6, 130.6, 130.0, 128.4, 125.3, 124.5, 122.3, 120.5, 116.8, 116.4, 115.1, 111.9, 9.6. MS-ESI (m/z) calcd. for [C_20_H_17_N_2_]^+^ : 285.1392, found : 285.1393.

**1-(2-phenylpyridin-4-yl)naphthalen-2-ol (5a)**. (26.8 mg, 90%). ^1^H NMR (400 MHz, Methylene Chloride-*d*_2_) δ 8.82 (dd, *J* = 4.9, 0.9 Hz, 1H), 8.11 – 8.01 (m, 2H), 7.87 (d, *J* = 8.8 Hz, 1H), 7.85 – 7.82 (m, 2H), 7.52 – 7.39 (m, 4H), 7.42 – 7.32 (m, 2H), 7.32 (dd, *J* = 4.9, 1.5 Hz, 1H), 7.27 (d, *J* = 8.9 Hz, 1H), 5.77 (br, 1H). ^13^C NMR (100 MHz, Methylene Chloride-*d*_2_) δ 158.5, 150.9, 150.7, 144.9, 139.2, 132.9, 130.7, 129.7, 129.3, 129.2, 128.5, 127.4, 125.1, 124.5, 124.0, 123.4, 119.3, 118.3. MS-ESI (m/z) calcd. for [C_21_H_16_NO]^+^ : 298.1232, found : 298.1233.

**6-methoxy-1-(2-phenylpyridin-4-yl)naphthalen-2-ol (5b)**. (29.0 mg, 89%). ^1^H NMR (400 MHz, DMSO-*d*_6_) δ 9.55 (s, 1H), 8.77 (d, *J* = 4.9 Hz, 1H), 8.19 – 8.05 (m, 2H), 7.89 (d, *J* = 1.2 Hz, 1H), 7.78 (d, *J* = 8.9 Hz, 1H), 7.52 – 7.46 (m, 2H), 7.46 – 7.41 (m, 1H), 7.33 (dd, *J* = 5.0, 1.5 Hz, 1H), 7.32 – 7.25 (m, 3H), 7.05 (dd, *J* = 9.3, 2.7 Hz, 1H), 3.84 (s, 3H). ^13^C NMR (100 MHz, DMSO-*d*_6_) δ 155.9, 155.1, 150.0, 149.3, 146.1, 138.6, 129.1, 128.9, 128.8, 128.5, 127.7, 126.6, 125.1, 125.0, 122.6, 119.1, 118.9, 118.7, 106.7, 55.2. MS-ESI (m/z) calcd. for [C_22_H_18_NO_2_]^+^ : 328.1338, found : 328.1339.

**7-methoxy-1-(2-phenylpyridin-4-yl)naphthalen-2-ol (5c)**. (32.2 mg, 98%). ^1^H NMR (400 MHz, DMSO-*d*_6_) δ 9.77 (s, 1H), 8.78 (dd, *J* = 4.9, 0.8 Hz, 1H), 8.17 – 8.08 (m, 2H), 7.94 (t, *J* = 1.2 Hz, 1H), 7.79 (dd, *J* = 8.9, 2.7 Hz, 2H), 7.50 (dd, *J* = 8.3, 6.3 Hz, 2H), 7.46 – 7.41 (m, 1H), 7.37 (dd, *J* = 4.9, 1.5 Hz, 1H), 7.15 (d, *J* = 8.8 Hz, 1H), 6.99 (dd, *J* = 8.9, 2.5 Hz, 1H), 6.74 (d, *J* = 2.5 Hz, 1H), 3.63 (s, 3H). ^13^C NMR (100 MHz, DMSO-*d*_6_) δ 158.0, 155.9, 152.5, 149.4, 146.2, 138.6, 133.8, 129.9, 129.6, 129.1, 128.8, 126.6, 125.1, 123.3, 122.6, 117.8, 115.7, 114.6, 102.7, 54.8.MS-ESI (m/z) calcd. for [C_22_H_18_NO_2_]^+^ : 328.1338, found : 328.1339.

**6-methyl-1-(2-phenylpyridin-4-yl)naphthalen-2-ol (5d)**. (30.9 mg, 99%). ^1^H NMR (400 MHz, DMSO-*d*_6_) δ 9.68 (s, 1H), 8.77 (d, *J* = 4.9 Hz, 1H), 8.19 – 8.08 (m, 2H), 7.89 (t, *J* = 1.2 Hz, 1H), 7.78 (d, *J* = 8.9 Hz, 1H), 7.64 (s, 1H), 7.49 (dd, *J* = 8.3, 6.2 Hz, 2H), 7.46 – 7.41 (m, 1H), 7.33 (dd, *J* = 5.0, 1.5 Hz, 1H), 7.32 – 7.26 (m, 2H), 7.21 (dd, *J* = 8.7, 1.8 Hz, 1H), 2.40 (s, 3H). ^13^C NMR (100 MHz, DMSO-*d*_6_) δ 155.9, 151.1, 149.4, 146.1, 138.7, 131.7, 130.7, 129.0, 129.0, 128.9, 128.8, 128.1, 127.0, 126.6, 125.1, 123.4, 122.6, 118.5, 118.3, 20.8. MS-ESI (m/z) calcd. for [C_22_H_18_NO]^+^ : 312.1388, found : 312.1389.

**3-methyl-1-(2-phenylpyridin-4-yl)naphthalen-2-ol (5e)**. (25.9 mg, 83%). ^1^H NMR (400 MHz, DMSO-*d*_6_) δ 8.80 (dd, *J* = 4.9, 0.8 Hz, 1H), 8.69 (s, 1H), 8.17 – 8.12 (m, 2H), 7.91 (t, *J* = 1.2 Hz, 1H), 7.82 – 7.78 (m, 1H), 7.77 (s, 1H), 7.52 – 7.47 (m, 2H), 7.46 – 7.40 (m, 1H), 7.34 – 7.23 (m, 4H), 2.42 (d, *J* = 1.0 Hz, 3H). ^13^C NMR (100 MHz, DMSO-*d*_6_) δ 156.1, 150.4, 149.6, 145.9, 138.7, 131.3, 129.1, 129.1, 128.8, 128.2, 127.8, 127.3, 126.6, 125.8, 125.3, 123.5, 123.1, 122.8, 120.0, 17.5. MS-ESI (m/z) calcd. for [C_22_H_18_NO]^+^ : 312.1388, found : 312.1389.

**6-bromo-1-(2-phenylpyridin-4-yl)naphthalen-2-ol (5f)**. (33.8 mg, 91%). ^1^H NMR (400 MHz, DMSO-*d*_6_) δ 10.03 (s, 1H), 8.78 (d, *J* = 5.0 Hz, 1H), 8.16 – 8.14 (m, 2H), 8.13 – 8.10 (m, 1H), 7.91 (t, *J* = 1.1 Hz, 1H), 7.88 (d, *J* = 9.0 Hz, 1H), 7.52 – 7.47 (m, 3H), 7.46 – 7.41 (m, 1H), 7.37 (d, *J* = 9.0 Hz, 1H), 7.35 – 7.30 (m, 2H). ^13^C NMR (100 MHz, DMSO-*d*_6_) δ 156.0, 152.3, 149.5, 145.4, 138.6, 131.2, 129.8, 129.6, 129.1, 129.1, 129.0, 128.8, 126.7, 125.9, 125.0, 122.6, 119.5, 118.8, 115.7. MS-ESI (m/z) calcd. for [C_21_H_15_BrNO]^+^ : 376.0337, found : 376.0337.

**6-chloro-1-(2-phenylpyridin-4-yl)naphthalen-2-ol (5g)**. (30.3 mg, 95%). ^1^H NMR (400 MHz, DMSO-*d*_6_) δ 10.01 (s, 1H), 8.79 (d, *J* = 4.9 Hz, 1H), 8.17 – 8.11 (m, 2H), 8.00 (t, *J* = 1.3 Hz, 1H), 7.92 (t, *J* = 1.1 Hz, 1H), 7.89 (d, *J* = 9.0 Hz, 1H), 7.52 – 7.47 (m, 2H), 7.46 – 7.42 (m, 1H), 7.42 – 7.36 (m, 3H), 7.34 (dd, *J* = 4.9, 1.5 Hz, 1H). ^13^C NMR (100 MHz, DMSO-*d*_6_) δ 156.0, 152.2, 149.5, 145.5, 138.6, 131.0, 129.1, 129.1, 128.8, 128.5, 127.3, 127.2, 126.7, 126.6, 125.8, 125.0, 122.6, 119.6, 118.8. MS-ESI (m/z) calcd. for [C_21_H_15_ClNO]^+^ : 332.0842, found : 332.0843.

**6-fluoro-1-(2-phenylpyridin-4-yl)naphthalen-2-ol (5h)**. (29.9 mg, 94%). ^1^H NMR (400 MHz, DMSO-*d*_6_) δ 9.84 (s, 1H), 8.78 (dd, *J* = 4.9, 0.8 Hz, 1H), 8.14 (d, *J* = 6.9 Hz, 1H), 7.92 (t, *J* = 1.2 Hz, 1H), 7.87 (d, *J* = 8.9 Hz, 1H), 7.68 (dd, *J* = 10.1, 2.7 Hz, 1H), 7.49 (dd, *J* = 8.3, 6.2 Hz, 2H), 7.47 – 7.39 (m, 2H), 7.37 (d, *J* = 9.0 Hz, 1H), 7.34 (dd, *J* = 5.0, 1.5 Hz, 1H), 7.28 (td, *J* = 9.0, 2.8 Hz, 1H). ^13^C NMR (100 MHz, DMSO-*d*_6_) δ 158.2 (d, *J* = 240.5 Hz), 156.0, 151.3 (d, *J* = 2.3 Hz), 149.5, 145.7, 138.6, 129.7, 129.1, 129.0 (d, *J* = 5.0 Hz), 128.8, 128.3 (d, *J* = 8.8 Hz), 126.7, 126.2 (d, *J* = 8.5 Hz), 125.1, 122.6, 119.6, 119.0, 116.6 (d, *J* = 25.0 Hz), 111.1 (d, *J* = 20.4 Hz). MS-ESI (m/z) calcd. for [C_21_H_15_FNO]^+^ : 316.1138, found : 316.1139.

**6-hydroxy-5-(2-phenylpyridin-4-yl)-2-naphthonitrile (5i)**. (31.0 mg, 95%). ^1^H NMR (400 MHz, DMSO-*d*_6_) δ 10.51 (s, 1H), 8.80 (d, *J* = 4.9 Hz, 1H), 8.53 (d, *J* = 1.7 Hz, 1H), 8.18 – 8.08 (m, 2H), 8.05 (d, *J* = 9.0 Hz, 1H), 7.93 (t, *J* = 1.1 Hz, 1H), 7.62 (dd, *J* = 8.9, 1.8 Hz, 1H), 7.52 – 7.41 (m, 5H), 7.35 (dd, *J* = 5.0, 1.5 Hz, 1H). ^13^C NMR (100 MHz, DMSO-*d*_6_) δ 156.1, 154.8, 149.6, 144.9, 138.5, 134.6, 134.3, 130.8, 129.2, 128.8, 127.3, 126.7, 125.0, 124.9, 122.5, 120.0, 119.4, 119.1, 104.9. MS-ESI (m/z) calcd. for [C_22_H_15_N_2_O]^+^ : 323.1184, found : 323.1185.

**6-hydroxy-5-(2-phenylpyridin-4-yl)-2-naphthamide (5j)**. (32.2 mg, 95%). ^1^H NMR (400 MHz, DMSO-*d*_6_) δ 10.14 (s, 1H), 8.80 (d, *J* = 4.9 Hz, 1H), 8.45 (d, *J* = 1.8 Hz, 1H), 8.17 – 8.10 (m, 2H), 8.03 (s, 1H), 7.97 (d, *J* = 8.9 Hz, 1H), 7.93 (s, 1H), 7.83 (dd, *J* = 8.9, 1.9 Hz, 1H), 7.49 (dd, *J* = 8.3, 6.2 Hz, 2H), 7.47 – 7.44 (m, 1H), 7.42 – 7.37 (m, 2H), 7.37 – 7.33 (m, 2H). ^13^C NMR (100 MHz, DMSO-*d*_6_) δ 167.9, 156.0, 153.3, 149.5, 145.7, 138.5, 133.9, 131.0, 129.1, 128.8, 128.5, 128.3, 126.9, 126.7, 125.3, 125.1, 123.3, 122.6, 119.0, 118.7. MS-ESI (m/z) calcd. for [C_22_H_17_N_2_O_2_]^+^ : 341.1290, found : 341.1289.

**methyl 6-hydroxy-5-(2-phenylpyridin-4-yl)-2-naphthoate (5k)**. (35.0 mg, 98%). ^1^H NMR (400 MHz, DMSO-*d*_6_) δ 10.32 (s, 1H), 8.80 (dd, *J* = 4.9, 0.8 Hz, 1H), 8.59 (d, *J* = 1.8 Hz, 1H), 8.17 – 8.08 (m, 3H), 7.94 (t, *J* = 1.2 Hz, 1H), 7.85 (dd, *J* = 8.9, 1.9 Hz, 1H), 7.52 – 7.44 (m, 4H), 7.42 (d, *J* = 8.9 Hz, 1H), 7.36 (dd, *J* = 4.9, 1.5 Hz, 1H), 3.88 (s, 3H). ^13^C NMR (100 MHz, DMSO-*d*_6_) δ 166.4, 156.1, 154.3, 149.5, 145.4, 138.5, 134.9, 131.6, 131.0, 129.1, 128.8, 126.8, 126.7, 125.8, 125.1, 124.0, 123.7, 122.6, 119.3, 118.8, 52.1. MS-ESI (m/z) calcd. for [C_23_H_18_NO_3_]^+^ : 356.1287, found : 356.1288.

**1-(6-hydroxy-5-(2-phenylpyridin-4-yl)naphthalen-2-yl)ethan-1-one (5l)**. (33.0 mg, 96%). ^1^H NMR (400 MHz, DMSO-*d*_6_) δ 10.33 (s, 1H), 8.80 (d, *J* = 4.9 Hz, 1H), 8.63 (d, *J* = 1.8 Hz, 1H), 8.15 – 8.12 (m, 2H), 8.11 (d, *J* = 9.0 Hz, 1H), 7.93 (s, 1H), 7.85 (dd, *J* = 8.9, 1.9 Hz, 1H), 7.52 – 7.40 (m, 5H), 7.36 (dd, *J* = 4.9, 1.5 Hz, 1H), 2.65 (s, 3H). ^13^C NMR (100 MHz, DMSO-*d*_6_) δ 197.4, 156.1, 154.4, 149.5, 145.5, 138.5, 134.9, 131.8, 131.4, 130.9, 129.2, 128.8, 126.8, 126.7, 125.1, 124.6, 123.8, 122.6, 119.1, 118.9, 26.6. MS-ESI (m/z) calcd. for [C_23_H_18_NO_2_]^+^ : 340.1338, found : 340.1339.

**6-hydroxy-5-(2-phenylpyridin-4-yl)-2-naphthaldehyde (5m)**. (31.0 mg, 95%). ^1^H NMR (400 MHz, DMSO-*d*_6_) δ 10.46 (s, 1H), 10.07 (s, 1H), 8.80 (dd, *J* = 5.0, 0.8 Hz, 1H), 8.54 (d, *J* = 1.7 Hz, 1H), 8.17 – 8.11 (m, 3H), 7.94 (t, *J* = 1.2 Hz, 1H), 7.77 (dd, *J* = 8.9, 1.7 Hz, 1H), 7.53 – 7.42 (m, 5H), 7.36 (dd, *J* = 4.9, 1.5 Hz, 1H). ^13^C NMR (100 MHz, DMSO-*d*_6_) δ 192.4, 156.1, 155.0, 149.5, 145.3, 138.5, 135.8, 135.0, 131.9, 131.2, 129.1, 128.8, 126.8, 126.7, 125.0, 124.5, 123.4, 122.6, 119.4, 119.4. MS-ESI (m/z) calcd. for [C_22_H_16_NO_2_]^+^ : 326.1181, found : 326.1182.

**6-(hydroxymethyl)-1-(2-phenylpyridin-4-yl)naphthalen-2-ol (5n)**. (29.0 mg, 87%). ^1^H NMR (400 MHz, DMSO-*d*_6_) δ 9.76 (s, 1H), 8.78 (d, *J* = 5.0 Hz, 1H), 8.13 (d, *J* = 6.8 Hz, 2H), 7.92 (s, 1H), 7.85 (d, *J* = 8.9 Hz, 1H), 7.78 (s, 1H), 7.50 (dd, *J* = 8.3, 6.2 Hz, 2H), 7.46 – 7.40 (m, 1H), 7.36 (td, *J* = 5.7, 3.1 Hz, 3H), 7.31 (dd, *J* = 9.0, 2.1 Hz, 1H), 5.21 (br, 1H), 4.60 (s, 2H). ^13^C NMR (100 MHz, DMSO-*d*_6_) δ 155.8, 151.5, 149.2, 146.3, 138.5, 136.9, 131.6, 129.6, 129.1, 128.8, 127.7, 126.7, 126.3, 125.2, 125.0, 123.3, 122.8, 118.5, 118.3, 62.8. MS-ESI (m/z) calcd. for [C_22_H_18_NO_2_]^+^ : 328.1332, found : 328.1338.

**5-(2-phenylpyridin-4-yl)quinolin-6-ol (5o)**. (16.9 mg, 58%). ^1^H NMR (400 MHz, DMSO-*d*_6_) δ 10.17 (s, 1H), 8.79 (d, *J* = 4.9 Hz, 1H), 8.72 (dd, *J* = 4.2, 1.6 Hz, 1H), 8.19 – 8.12 (m, 2H), 8.00 (d, *J* = 9.1 Hz, 1H), 7.94 (s, 1H), 7.82 (dd, *J* = 8.7, 1.6 Hz, 1H), 7.57 (d, *J* = 9.1 Hz, 1H), 7.52 – 7.46 (m, 2H), 7.46 – 7.42 (m, 1H), 7.39 (dd, *J* = 8.6, 4.1 Hz, 1H), 7.36 (dd, *J* = 4.9, 1.5 Hz, 1H). ^13^C NMR (100 MHz, DMSO-*d*_6_) δ 156.1, 152.1, 149.6, 147.1, 144.7, 142.9, 138.6, 131.8, 130.6, 128.8, 127.5, 126.7, 125.0, 122.5, 121.9, 121.7, 118.4. MS-ESI (m/z) calcd. for [C_20_H_15_N_2_O]^+^ : 299.1184, found : 299.1185.

**8-(2-phenylpyridin-4-yl)isoquinolin-7-ol (5p)**. (26.0 mg, 85%). ^1^H NMR (400 MHz, DMSO-*d*_6_) δ 10.28 (s, 1H), 8.88 – 8.63 (m, 2H), 8.37 (br, 1H), 8.24 – 8.14 (m, 2H), 8.00 (s, 1H), 7.97 (d, *J* = 8.9 Hz, 1H), 7.81 (d, *J* = 5.3 Hz, 1H), 7.59 (d, *J* = 8.9 Hz, 1H), 7.50 (dd, *J* = 8.3, 6.2 Hz, 2H), 7.47 – 7.44 (m, 1H), 7.42 (dd, *J* = 4.9, 1.5 Hz, 1H). ^13^C NMR (100 MHz, DMSO-*d*_6_) δ 156.1, 152.8, 149.6, 147.9, 144.0, 139.9, 138.6, 130.1, 129.1, 128.8, 128.6, 126.7, 125.0, 123.1, 122.5, 120.6, 118.5. MS-ESI (m/z) calcd. for [C_20_H_15_N_2_O]^+^ : 299.1184, found : 299.1184.

**4-(2-phenylpyridin-4-yl)benzofuran-5-ol (5q)**. (7.6 mg, 26%). ^1^H NMR (400 MHz, Methanol-*d*_4_) δ 8.68 (d, *J* = 5.2 Hz, 1H), 8.09 – 8.05 (m, 1H), 7.98 – 7.92 (m, 2H), 7.75 (d, *J* = 2.2 Hz, 1H), 7.64 (dd, *J* = 5.3, 1.6 Hz, 1H), 7.55 – 7.50 (m, 2H), 7.49 – 7.44 (m, 1H), 7.42 (dd, *J* = 8.9, 0.9 Hz, 1H), 6.96 (d, *J* = 8.8 Hz, 1H), 6.78 (dd, *J* = 2.3, 0.9 Hz, 1H). ^13^C NMR (100 MHz, Methanol-*d*_4_) δ 158.6, 151.4, 151.0, 149.5, 148.7, 148.1, 140.2, 130.4, 130.0, 128.7, 128.3, 125.2, 124.3, 117.7, 114.8, 113.5, 106.6. MS-ESI (m/z) calcd. for [C_19_H_14_NO_2_]^+^ : 288.1025, found : 288.1025.

**4-(2-phenylpyridin-4-yl)benzo[b]thiophen-5-ol (5r)**. (14.6 mg, 50%). ^1^H NMR (400 MHz, Methanol-*d*_4_) δ 8.68 (dd, *J* = 5.2, 0.8 Hz, 1H), 7.97 – 7.90 (m, 3H), 7.77 (dd, *J* = 8.7, 0.8 Hz, 1H), 7.56 – 7.47 (m, 4H), 7.47 – 7.41 (m, 1H), 7.12 (dd, *J* = 5.6, 0.8 Hz, 1H), 7.06 (d, *J* = 8.7 Hz, 1H). ^13^C NMR (100 MHz, Methanol-*d*_4_) δ 158.7, 152.6, 149.7, 149.1, 140.7, 140.3, 133.2, 130.3, 129.9, 129.4, 128.3, 125.8, 124.8, 124.6, 123.1, 120.6, 115.9. MS-ESI (m/z) calcd. for [C_19_H_14_NOS]^+^ : 304.0796, found : 304.0797.

**1-(pyridin-4-yl)naphthalen-2-ol (5s)**. (6.0 mg, 24%). ^1^H NMR (400 MHz, DMSO-*d*_6_) δ 9.83 (s, 1H), 8.84 – 8.58 (m, 2H), 7.88 – 7.82 (m, 2H), 7.40 – 7.37 (m, 2H), 7.35 (dd, *J* = 6.7, 2.1 Hz, 1H), 7.30 (dd, *J* = 8.5, 5.6 Hz, 3H). ^13^C NMR (100 MHz, DMSO-*d*_6_) δ 151.7, 149.3, 145.1, 132.4, 129.8, 128.2, 127.8, 126.8, 126.5, 123.3, 122.8, 118.3, 118.3. MS-ESI (m/z) calcd. for [C_15_H_12_NO]^+^ : 222.1031, found : 222.1030.

**1-(2-(4-methoxyphenyl)pyridin-4-yl)naphthalen-2-ol (5t)**. (23.3 mg, 71%). ^1^H NMR (400 MHz, DMSO-*d*_6_) δ 9.80 (s, 1H), 8.72 (d, *J* = 4.9 Hz, 1H), 8.09 (d, *J* = 8.9 Hz, 2H), 7.93 – 7.79 (m, 3H), 7.39 – 7.35 (m, 2H), 7.35 – 7.28 (m, 2H), 7.26 (dd, *J* = 5.0, 1.5 Hz, 1H), 7.07 – 7.00 (m, 2H), 3.81 (s, 3H). ^13^C NMR (100 MHz, DMSO-*d*_6_) δ 160.2, 155.7, 151.7, 149.2, 145.9, 132.5, 131.1, 129.7, 128.1, 128.0, 127.8, 126.8, 124.4, 123.5, 122.8, 121.8, 118.7, 118.3, 114.1, 55.2. MS-ESI (m/z) calcd. for [C_22_H_18_NO_2_]^+^ : 328.1338, found : 328.1339.

**1-(2-(4-(trifluoromethyl)phenyl)pyridin-4-yl)naphthalen-2-ol (5u)**. (33.2 mg, 91%). ^1^H NMR (400 MHz, DMSO-*d*_6_) δ 9.86 (s, 1H), 8.84 (d, *J* = 4.9 Hz, 1H), 8.37 (d, *J* = 8.2 Hz, 2H), 8.05 (s, 1H), 7.89 (dd, *J* = 8.4, 5.0 Hz, 2H), 7.84 (d, *J* = 8.3 Hz, 2H), 7.44 (dd, *J* = 4.9, 1.5 Hz, 1H), 7.38 (dd, *J* = 3.6, 1.2 Hz, 2H), 7.35 – 7.29 (m, 2H). ^13^C NMR (100 MHz, DMSO-*d*_6_) δ 154.4, 151.8, 149.7, 146.4, 142.5, 132.5, 129.9, 129.2 (q, *J* = 31.9 Hz), 128.2, 127.8, 127.4, 126.9, 126.1, 125.7 (q, *J* = 3.7 Hz), 123.4, 123.4, 124.3 (q, *J* = 272.1 Hz), 122.8, 118.3, 118.3. MS-ESI (m/z) calcd. for [C_22_H_15_F_3_NO]^+^ : 366.1106, found : 366.1107.

**1-(2-(4-bromophenyl)pyridin-4-yl)naphthalen-2-ol (5v)**. (35.0 mg, 94%). ^1^H NMR (400 MHz, DMSO-*d*_6_) δ 9.83 (s, 1H), 8.78 (dd, *J* = 5.0, 0.8 Hz, 1H), 8.15 – 8.07 (m, 2H), 7.95 (s, 1H), 7.90 – 7.83 (m, 2H), 7.68 (d, *J* = 8.6 Hz, 2H), 7.39 – 7.36 (m, 3H), 7.35 – 7.28 (m, 2H). ^13^C NMR (100 MHz, DMSO-*d*_6_) δ 154.8, 151.8, 149.5, 146.2, 137.8, 132.5, 131.7, 129.8, 128.7, 128.2, 127.8, 126.8, 125.5, 123.4, 122.8, 122.7, 118.4, 118.3. MS-ESI (m/z) calcd. for [C_21_H_15_BrNO]^+^ : 376.0337, found : 376.0338.

**1-(2-(thiophen-2-yl)pyridin-4-yl)naphthalen-2-ol (5w)**. (24.1 mg, 83%). ^1^H NMR (400 MHz, DMSO-*d*_6_) δ 9.82 (s, 1H), 8.63 (dd, *J* = 5.0, 0.8 Hz, 1H), 7.92 – 7.83 (m, 3H), 7.82 (dd, *J* = 3.7, 1.1 Hz, 1H), 7.64 (dd, *J* = 5.0, 1.1 Hz, 1H), 7.42 – 7.27 (m, 4H), 7.24 (dd, *J* = 5.0, 1.5 Hz, 1H), 7.14 (dd, *J* = 5.1, 3.7 Hz, 1H). ^13^C NMR (100 MHz, DMSO-*d*_6_) δ 151.9, 151.7, 149.2, 146.0, 144.7, 132.5, 129.7, 128.4, 128.4, 128.1, 127.8, 126.8, 125.3, 124.9, 123.4, 122.8, 120.9, 118.4, 118.3. MS-ESI (m/z) calcd. for [C_19_H_14_NOS]^+^ : 304.0796, found : 304.0797.

**1-([2,2'-bipyridin]-4-yl)naphthalen-2-ol (5x)**. (26.0 mg, 86%). ^1^H NMR (400 MHz, Methanol-*d*_4_) δ 8.76 (dd, *J* = 5.0, 0.8 Hz, 1H), 8.61 (ddd, *J* = 4.9, 1.7, 0.9 Hz, 1H), 8.37 (dt, *J* = 8.0, 1.1 Hz, 1H), 8.32 (dd, *J* = 1.5, 0.9 Hz, 1H), 7.96 (td, *J* = 7.8, 1.8 Hz, 1H), 7.84 – 7.78 (m, 2H), 7.48 (dd, *J* = 5.0, 1.6 Hz, 1H), 7.46 – 7.40 (m, 2H), 7.36 – 7.27 (m, 2H), 7.24 (d, *J* = 8.9 Hz, 1H). ^13^C NMR (100 MHz, Methanol-*d*_4_) δ 157.3, 157.1, 152.9, 150.3, 150.2, 148.6, 138.8, 134.2, 131.2, 130.0, 129.3, 128.3, 127.8, 125.6, 125.3, 124.7, 124.1, 122.9, 120.1, 119.0. MS-ESI (m/z) calcd. for [C_20_H_15_N_2_O]^+^ : 299.1184, found : 299.1185.

**methyl 4-(2-hydroxynaphthalen-1-yl)picolinate (5y)**. White solid (24 mg, 85%). ^1^H NMR (400 MHz, DMSO-*d*_6_) δ 9.94 (s, 1H), 8.83 (d, *J* = 4.9 Hz, 1H), 8.00 (d, *J* = 1.6 Hz, 1H), 7.92 – 7.84 (m, 2H), 7.65 (dd, *J* = 4.9, 1.7 Hz, 1H), 7.41 – 7.28 (m, 4H), 3.90 (s, 3H). ^13^C NMR (100 MHz, DMSO-*d*_6_) δ 165.4, 151.9, 149.9, 147.6, 146.0, 132.2, 130.3, 129.8, 128.3, 127.8, 127.3, 127.0, 123.0, 122.9, 118.2, 117.4, 52.5. MS-ESI (m/z) calcd. for [C_17_H_14_NO_3_]^+^ : 280.0968, found : 280.0974.

**1-(2-phenylquinolin-4-yl)naphthalen-2-ol (5z)**. White solid (32 mg, 92%). ^1^H NMR (400 MHz, DMSO-*d*_6_) δ 9.79 (s, 1H), 8.34 (dd, *J* = 8.2, 1.6 Hz, 2H), 8.20 (dd, *J* = 8.5, 1.1 Hz, 1H), 8.06 (s, 1H), 7.99 (d, *J* = 8.8 Hz, 1H), 7.92 (dd, *J* = 8.1, 1.5 Hz, 1H), 7.78 (ddd, *J* = 8.4, 6.8, 1.5 Hz, 1H), 7.58 – 7.47 (m, 3H), 7.45 – 7.36 (m, 2H), 7.33 – 7.22 (m, 3H), 7.01 (dd, *J* = 8.4, 1.3 Hz, 1H). ^13^C NMR (100 MHz, DMSO-*d*_6_) δ 155.8, 152.4, 147.9, 145.2, 138.5, 133.3, 130.0, 129.9, 129.7, 129.5, 128.9, 128.1, 127.8, 127.3, 126.9, 126.8, 126.5, 125.7, 123.8, 122.8, 120.9, 118.3, 116.4. MS-ESI (m/z) calcd. for [C_25_H_18_NO]^+^ : 348.1383, found : 348.1388.

**2-chloro-N-(4-chloro-3-(4-(2-hydroxynaphthalen-1-yl)pyridin-2-yl)phenyl)-4-(methylsulfonyl)benzamide (5aa)**. (43.2 mg, 77%). ^1^H NMR (400 MHz, Methylene Chloride-*d*_2_) δ 9.43 (s, 1H), 8.63 (d, *J* = 5.1 Hz, 1H), 7.94 – 7.86 (m, 3H), 7.82 (dd, *J* = 8.6, 2.1 Hz, 2H), 7.78 – 7.73 (m, 2H), 7.60 (d, *J* = 8.0 Hz, 1H), 7.50 – 7.44 (m, 2H), 7.42 – 7.32 (m, 3H), 7.21 (d, *J* = 8.9 Hz, 1H), 2.99 (s, 3H). ^13^C NMR (100 MHz, Methylene Chloride-*d*_2_) δ 164.2, 156.4, 151.0, 149.3, 145.7, 143.3, 140.8, 138.7, 137.5, 132.8, 132.5, 131.3, 131.0, 130.5, 129.4, 129.3, 128.6, 128.5, 127.9, 127.6, 126.4, 126.2, 124.2, 124.1, 123.4, 122.2, 118.8, 118.4, 44.6. MS-ESI (m/z) calcd. for [C_29_H_21_Cl_2_N_2_O_4_S]^+^ : 563.0599, found : 563.0599.

***Appendix I***

**Spectral Copies of 1H-, 13C-Data**

**3-(2-phenylpyridin-4-yl)-1H-indole (3a)**.

**400MHz, ^1^H NMR in DMSO-*d*_6_**

**100 MHz, ^13^C NMR in DMSO-*d*_6_**

**5-methoxy-3-(2-phenylpyridin-4-yl)-1H-indole (3b)**.

**400MHz, ^1^H NMR in Methylene Chloride-*d*_2_**

**100 MHz, ^13^C NMR in Methylene Chloride-*d*_2_**

**5-methyl-3-(2-phenylpyridin-4-yl)-1H-indole (3c)**.

**400MHz, ^1^H NMR in DMSO-*d*_6_**

**100 MHz, ^13^C NMR in DMSO-*d*_6_**

**7-methyl-3-(2-phenylpyridin-4-yl)-1H-indole (3d)**.

**400MHz, ^1^H NMR in DMSO-*d*_6_**

**100 MHz, ^13^C NMR in DMSO-*d*_6_**

**2-methyl-3-(2-phenylpyridin-4-yl)-1H-indole (3e)**.

**400MHz, ^1^H NMR in DMSO-*d*_6_**

**100 MHz, ^13^C NMR in DMSO-*d*_6_**

**5-iodo-3-(2-phenylpyridin-4-yl)-1H-indole (3f)**.

**400MHz, ^1^H NMR in DMSO-*d*_6_**

**100 MHz, ^13^C NMR in DMSO-*d*_6_**

**5-bromo-3-(2-phenylpyridin-4-yl)-1H-indole (3g)**.

**400MHz, ^1^H NMR in DMSO-*d*_6_**

**100 MHz, ^13^C NMR in DMSO-*d*_6_**

**5-chloro-3-(2-phenylpyridin-4-yl)-1H-indole (3h)**.

**400MHz, ^1^H NMR in DMSO-*d*_6_**

**100 MHz, ^13^C NMR in DMSO-*d*_6_**

**5-fluoro-3-(2-phenylpyridin-4-yl)-1H-indole (3i)**.

**400MHz, ^1^H NMR in DMSO-*d*_6_**

**100 MHz, ^13^C NMR in DMSO-*d*_6_**

**5-nitro-3-(2-phenylpyridin-4-yl)-1H-indole (3j)**.

**400MHz, ^1^H NMR in DMSO-*d*_6_**

**100 MHz, ^13^C NMR in DMSO-*d*_6_**

**3-(2-phenylpyridin-4-yl)-1H-indole-5-carbonitrile (3k)**.

**400MHz, ^1^H NMR in DMSO-*d*_6_**

**100 MHz, ^13^C NMR in DMSO-*d*_6_**

**3-(2-phenylpyridin-4-yl)-1H-indole-5-carboxamide (3l)**.

**400MHz, ^1^H NMR in DMSO-*d*_6_**

**100 MHz, ^13^C NMR in DMSO-*d*_6_**

**3-(2-phenylpyridin-4-yl)-1H-indole-6-carbaldehyde (3m)**.

**400MHz, ^1^H NMR in DMSO-*d*_6_**

**100 MHz, ^13^C NMR in DMSO-*d*_6_**

**(3-(2-phenylpyridin-4-yl)-1H-indol-6-yl)methanol (3n)**.

**400MHz, ^1^H NMR in DMSO-*d*_6_**

**100 MHz, ^13^C NMR in DMSO-*d*_6_**

**3-(2-phenylpyridin-4-yl)-1H-pyrrolo[2,3-b]pyridine (3o)**.

**400MHz, ^1^H NMR in DMSO-*d*_6_**

**100 MHz, ^13^C NMR in DMSO-*d*_6_**

**5-(2-phenylpyridin-4-yl)-7H-pyrrolo[2,3-d]pyrimidine (3p)**.

**400MHz, ^1^H NMR in DMSO-*d*_6_**

**100 MHz, ^13^C NMR in DMSO-*d*_6_**

**5-chloro-3-(pyridin-4-yl)-1H-indole (3q)**.

**400MHz, ^1^H NMR in DMSO-*d*_6_**

**100 MHz, ^13^C NMR in DMSO-*d*_6_**

**5-chloro-3-(2-(4-methoxyphenyl)pyridin-4-yl)-1H-indole (3r)**.

**400MHz, ^1^H NMR in DMSO-*d*_6_**

**100 MHz, ^13^C NMR in DMSO-*d*_6_**

**5-chloro-3-(2-(4-(trifluoromethyl)phenyl)pyridin-4-yl)-1H-indole (3s)**.

**400MHz, ^1^H NMR in DMSO-*d*_6_**

**100 MHz, ^13^C NMR in DMSO-*d*_6_**

**3-(2-(4-bromophenyl)pyridin-4-yl)-5-chloro-1H-indole (3t)**.

**400MHz, ^1^H NMR in DMSO-*d*_6_**

**100 MHz, ^13^C NMR in DMSO-*d*_6_**

**5-chloro-3-(2-(thiophen-2-yl)pyridin-4-yl)-1H-indole (3u)**.

**400MHz, ^1^H NMR in DMSO-*d*_6_**

**100 MHz, ^13^C NMR in DMSO-*d*_6_**

**3-([2,2'-bipyridin]-4-yl)-5-chloro-1H-indole (3v)**.

**400MHz, ^1^H NMR in Methylene Chloride-*d*_2_**

**100 MHz, ^13^C NMR in Methylene Chloride-*d*_2_**

**methyl 4-(5-chloro-1H-indol-3-yl)picolinate (3w)**.

**400MHz, ^1^H NMR in DMSO-*d*_6_**

**100 MHz, ^13^C NMR in DMSO-*d*_6_**

**4-(5-chloro-1H-indol-3-yl)-2-phenylquinoline (3x)**.

**400MHz, ^1^H NMR in DMSO-*d*_6_**

**100 MHz, ^13^C NMR in DMSO-*d*_6_**

**2-chloro-N-(4-chloro-3-(4-(6-formyl-1H-indol-3-yl)pyridin-2-yl)phenyl)-4-(methylsulfonyl)benzamide (3y)**.

**400MHz, ^1^H NMR in DMSO-*d*_6_**

**100 MHz, ^13^C NMR in DMSO-*d*_6_**

**3-methyl-1-(2-phenylpyridin-4-yl)-1H-indole (3z)**.

**400MHz, ^1^H NMR in Methanol-*d*_4_**

**400MHz, ^13^C NMR in Methanol-*d*_4_**

**1-(2-phenylpyridin-4-yl)naphthalen-2-ol (5a)**.

**400MHz, ^1^H NMR in Methylene Chloride-*d*_2_**

**100 MHz, ^13^C NMR in Methylene Chloride-*d*_2_**

**6-methoxy-1-(2-phenylpyridin-4-yl)naphthalen-2-ol (5b)**.

**400MHz, ^1^H NMR in DMSO-*d*_6_**

**100 MHz, ^13^C NMR in DMSO-*d*_6_**

**7-methoxy-1-(2-phenylpyridin-4-yl)naphthalen-2-ol (5c)**.

**400MHz, ^1^H NMR in DMSO-*d*_6_**

**100 MHz, ^13^C NMR in DMSO-*d*_6_**

**6-methyl-1-(2-phenylpyridin-4-yl)naphthalen-2-ol (5d)**.

**400MHz, ^1^H NMR in DMSO-*d*_6_**

**100 MHz, ^13^C NMR in DMSO-*d*_6_**

**3-methyl-1-(2-phenylpyridin-4-yl)naphthalen-2-ol (5e)**.

**400MHz, ^1^H NMR in DMSO-*d*_6_**

**100 MHz, ^13^C NMR in DMSO-*d*_6_**

**6-bromo-1-(2-phenylpyridin-4-yl)naphthalen-2-ol (5f)**.

**400MHz, ^1^H NMR in DMSO-*d*_6_**

**100 MHz, ^13^C NMR in DMSO-*d*_6_**

**6-chloro-1-(2-phenylpyridin-4-yl)naphthalen-2-ol (5g)**.

**400MHz, ^1^H NMR in DMSO-*d*_6_**

**100 MHz, ^13^C NMR in DMSO-*d*_6_**

**6-fluoro-1-(2-phenylpyridin-4-yl)naphthalen-2-ol (5h)**.

**400MHz, ^1^H NMR in DMSO-*d*_6_**

**100 MHz, ^13^C NMR in DMSO-*d*_6_**

**6-hydroxy-5-(2-phenylpyridin-4-yl)-2-naphthonitrile (5i)**.

**400MHz, ^1^H NMR in DMSO-*d*_6_**

**100 MHz, ^13^C NMR in DMSO-*d*_6_**

**6-hydroxy-5-(2-phenylpyridin-4-yl)-2-naphthamide (5j)**.

**400MHz, ^1^H NMR in DMSO-*d*_6_**

**100 MHz, ^13^C NMR in DMSO-*d*_6_**

**methyl 6-hydroxy-5-(2-phenylpyridin-4-yl)-2-naphthoate (5k)**.

**400MHz, ^1^H NMR in DMSO-*d*_6_**

**100 MHz, ^13^C NMR in DMSO-*d*_6_**

**1-(6-hydroxy-5-(2-phenylpyridin-4-yl)naphthalen-2-yl)ethan-1-one (5l)**.

**400MHz, ^1^H NMR in DMSO-*d*_6_**

**100 MHz, ^13^C NMR in DMSO-*d*_6_**

**6-hydroxy-5-(2-phenylpyridin-4-yl)-2-naphthaldehyde (5m)**.

**400MHz, ^1^H NMR in DMSO-*d*_6_**

**100 MHz, ^13^C NMR in DMSO-*d*_6_**

**6-(hydroxymethyl)-1-(2-phenylpyridin-4-yl)naphthalen-2-ol (5n)**.

**400MHz, ^1^H NMR in DMSO-*d*_6_**

**100 MHz, ^13^C NMR in DMSO-*d*_6_**

**5-(2-phenylpyridin-4-yl)quinolin-6-ol (5o)**.

**400MHz, ^1^H NMR in DMSO-*d*_6_**

**100 MHz, ^13^C NMR in DMSO-*d*_6_**

**8-(2-phenylpyridin-4-yl)isoquinolin-7-ol (5p)**.

**400MHz, ^1^H NMR in DMSO-*d*_6_**

**100 MHz, ^13^C NMR in DMSO-*d*_6_**

**4-(2-phenylpyridin-4-yl)benzofuran-5-ol (5q)**.

**400MHz, ^1^H NMR in Methanol-*d*_4_**

**100 MHz, ^13^C NMR in Methanol-*d*_4_**

**4-(2-phenylpyridin-4-yl)benzo[b]thiophen-5-ol (5r)**.

**400MHz, ^1^H NMR in Methanol-*d*_4_**

**100 MHz, ^13^C NMR in Methanol-*d*_4_**

**1-(pyridin-4-yl)naphthalen-2-ol (5s)**.

**400MHz, ^1^H NMR in DMSO-*d*_6_**

**100 MHz, ^13^C NMR in DMSO-*d*_6_**

**1-(2-(4-methoxyphenyl)pyridin-4-yl)naphthalen-2-ol (5t)**.

**400MHz, ^1^H NMR in DMSO-*d*_6_**

**100 MHz, ^13^C NMR in DMSO-*d*_6_**

**1-(2-(4-(trifluoromethyl)phenyl)pyridin-4-yl)naphthalen-2-ol (5u)**.

**400MHz, ^1^H NMR in DMSO-*d*_6_**

**100 MHz, ^13^C NMR in DMSO-*d*_6_**

**1-(2-(4-bromophenyl)pyridin-4-yl)naphthalen-2-ol (5v)**.

**400MHz, ^1^H NMR in DMSO-*d*_6_**

**100 MHz, ^13^C NMR in DMSO-*d*_6_**

**1-(2-(thiophen-2-yl)pyridin-4-yl)naphthalen-2-ol (5w)**.

**400MHz, ^1^H NMR in DMSO-*d*_6_**

**100 MHz, ^13^C NMR in DMSO-*d*_6_**

**1-([2,2'-bipyridin]-4-yl)naphthalen-2-ol (5x)**.

**400MHz, ^1^H NMR in Methanol-*d*_4_**

**100 MHz, ^13^C NMR in Methanol-*d*_4_**

**methyl 4-(2-hydroxynaphthalen-1-yl)picolinate (5y)**.

**400MHz, ^1^H NMR in DMSO-*d*_6_**

**100 MHz, ^13^C NMR in DMSO-*d*_6_**

**1-(2-phenylquinolin-4-yl)naphthalen-2-ol (5z)**.

**400MHz, ^1^H NMR in DMSO-*d*_6_**

**100 MHz, ^13^C NMR in DMSO-*d*_6_**

**2-chloro-N-(4-chloro-3-(4-(2-hydroxynaphthalen-1-yl)pyridin-2-yl)phenyl)-4-(methylsulfonyl)benzamide (5aa)**.

**400MHz, ^1^H NMR in Methylene Chloride-*d*_2_**

**100 MHz, ^13^C NMR in Methylene Chloride-*d*_2_**
